# Leonurine microcomposite depots alleviate endometriosis and rheumatoid arthritis *via* shared anti-inflammatory cascades

**DOI:** 10.1016/j.ijpx.2026.100690

**Published:** 2026-10-17

**Authors:** Shiyu Meng, Jingyi Zhong, Yi Xiao, Xican Zhang, Zhuang Tang, Zhiling Song, Xiaoxue Yang, Yi Zhun Zhu, Xiaolin Wang, Hui Guo

**Affiliations:** School of Pharmacy and State Key Laboratory of Mechanism and Quality of Chinese Medicine, Macau University of Science and Technology, Macao, 999078, China

**Keywords:** Endometriosis, Rheumatoid arthritis, Leonurine, Sustained release, Anti-inflammation, Autophagy

## Abstract

Chronic inflammatory diseases, such as endometriosis (EMS) and rheumatoid arthritis (RA), share a common pathogenic hallmark driven by a hyperinflammatory microenvironment. Nonetheless, long-acting therapeutic platforms capable of concurrently addressing these disparate disorders remain rarely explored. Herein, we developed a leonurine microcrystal-encapsulated microcomposite depot (Leo-micro@MD) capable of effectively mitigating inflammation, thereby reversing the progression of both EMS and RA. Systemic formulation optimization yielded an optimized Leo-micro@MD formulation with high drug loading and a sustained, burst-free release profile extending over 18 days, markedly reducing administration frequency. Mechanistically, Leo-micro@MD suppresses key upstream inflammatory cytokines, thereby branching into distinct downstream therapeutic pathways tailored to each disorder. In EMS, this reduction attenuated estrogen-ERα signaling and restored progesterone receptor B (PRB) expression, subsequently reactivating LC3B-mediated autophagy and Bax/Bcl-2-dependent apoptosis to eradicate ectopic lesions. Concurrently, in an adjuvant-induced arthritis rat model, this anti-inflammatory cascade attenuates synovial hyperplasia, rescues bone mineral density and trabecular architecture, and re-establishes articular matrix homeostasis, as confirmed by micro-CT and comprehensive histopathology. Importantly, these disease-modifying effects are achieved with an excellent systemic safety profile, with no detectable hepatotoxicity, nephrotoxicity, or immune organ damage. Collectively, this study provides a robust and translatable controlled-release strategy for the comprehensive management of chronic inflammatory diseases.

## Graphical abstract

Leo-micro@MD achieves sustained drug release to suppress a shared chronic inflammatory hub, concurrently driving ectopic lesion regression in endometriosis and preserving cartilage and bone integrity in rheumatoid arthritis.

**Figure:**
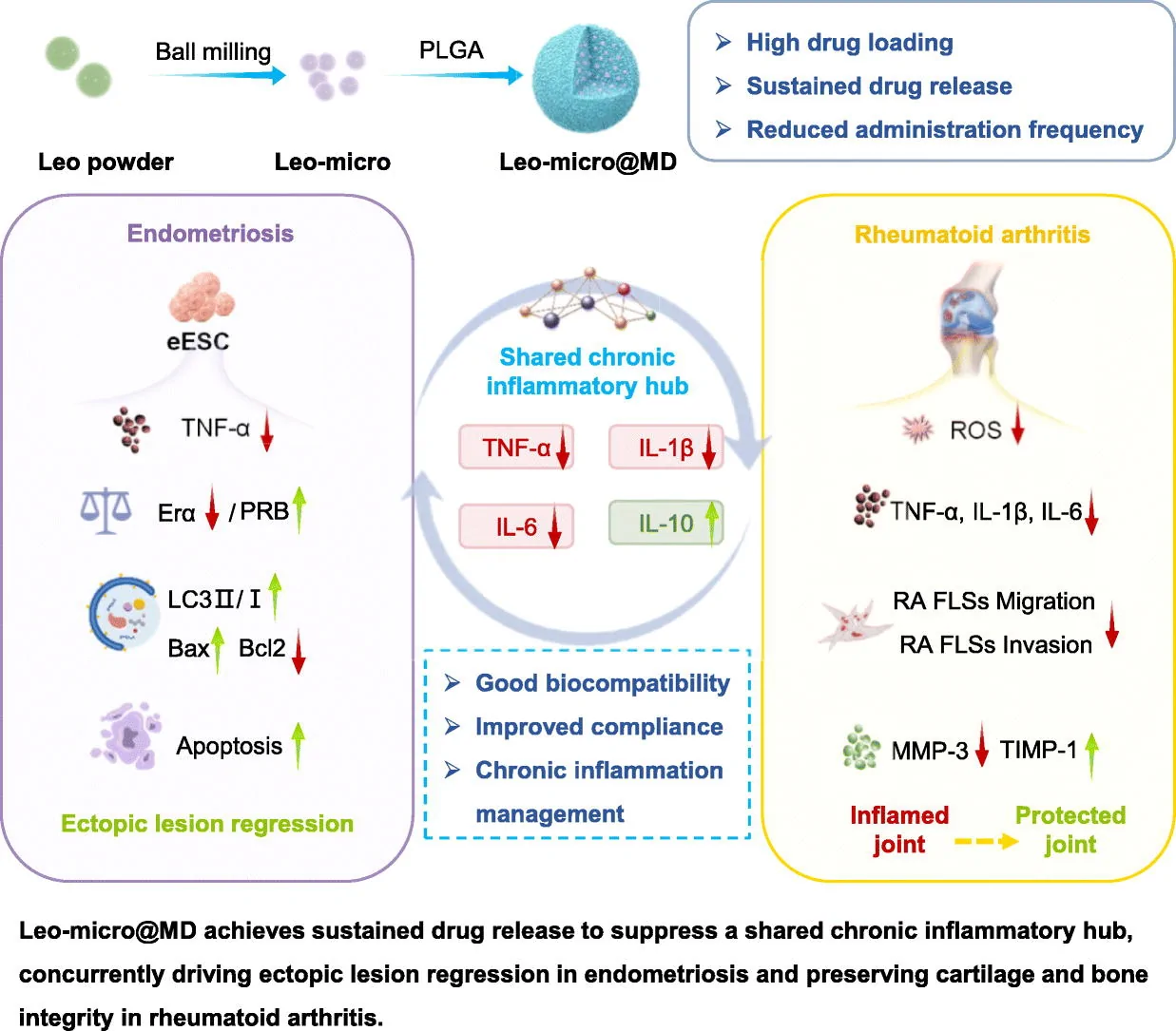

## 1 Introduction

Inflammation serves as a pivotal homeostatic mechanism orchestrating tissue repair, but its persistence transitions into a chronic state characterized by the aberrant secretion of pro-inflammatory mediators (Mao et al., 2025). Among these, pro-inflammatory cytokines, such as tumor necrosis factor-alpha (TNF-α), serve as cornerstone hubs that drive downstream tissue remodeling and immunological dysregulation. Crucially, the hyperinflammatory microenvironment underpins the pathogenesis of seemingly disparate chronic disorders, notably endometriosis (EMS) and rheumatoid arthritis (RA) (Alivernini et al., 2022; As-Sanie et al., 2025). EMS is an estrogen-dependent reproductive disorder where ectopic endometrial tissue implants outside the uterine cavity, stimulating pelvic pain and infertility *via* localized TNF-α accumulation (Greenhill, 2024). Parallelly, RA manifests as an immune-mediated arthropathy where elevated TNF-α activates fibroblast-like synoviocytes (FLS), leading to irreversible bone degradation (Komatsu and Takayanagi, 2022). Clinical evidence indicates that patients with EMS possess an elevated susceptibility to RA, supported by significant genetic overlaps in cytokine variants (Harris et al., 2016; Wei et al., 2020; Zervou et al., 2022). These data collectively reframe EMS and RA as manifestations of a systemic chronic inflammatory process, rather than as site-restricted pathologies.

Contemporary clinical interventions for EMS and RA remain constrained to symptom-relieving hormonal therapies or non-steroidal anti-inflammatory drugs, whose prolonged use triggers severe gastrointestinal and cardiovascular toxicities (Brown et al., 2024; Ye et al., 2022). To circumvent these constraints, Leonurine (Leo), a unique alkaloid isolated from Herba Leonuri, has emerged as a promising therapeutic candidate due to its potent anti-inflammatory properties and is currently undergoing clinical translation by our research group (Tang et al., 2023; Tang et al., 2024a). Mechanistically, Leo can rectify the aberrant inflammatory endocrine-autophagy axis in EMS by suppressing the TNF-α pathway, thereby upregulating progesterone receptor expression and inducing apoptosis in ectopic lesions (Li et al., 2022; Tang et al., 2024b). In parallel, Leo can mitigate RA synovial inflammation by neutralizing FLS activation (Meng et al., 2023; Tang et al., 2024a). Nevertheless, the clinical translation of free Leo is impeded by its poor water solubility and rapid *in vivo* clearance, necessitating frequent, high-dose administrations.

Consequently, developing long-acting drug delivery systems is imperative to achieve sustained therapeutic interventions of Leo for chronic inflammatory-dominated disorders (Li et al., 2026). Compared with nano-sized delivery platforms such as liposomes, which primarily excel in cellular targeting or acute localization, micron-sized spherical matrices offer unique advantages as localized long-acting depots (Song et al., 2023). These formulations can uniformly encapsulate therapeutic payloads and govern their release profiles through predictable, extended matrix erosion, effectively translating daily systemic dosing into weekly intervals to enhance patient compliance (Lin et al., 2024). Among available biomaterials, poly(lactic-*co*-glycolide) (PLGA) copolymers are exceptionally suited due to their established biocompatibility and adjustable degradation rates, offering a powerful platform for sustained chronic disease management (Wan et al., 2023).

In this work, we introduce a unified therapeutic strategy for managing distinct chronic inflammatory diseases through a single long-acting platform. To this end, we co-encapsulated leonurine microcrystals (Leo-micro) and magnesium carbonate (MgCO₃) within a PLGA matrix *via* a solid-in-oil-in-water emulsion-solvent evaporation method, yielding the microcomposite depot (Leo-micro@MD) (Scheme 1). Notably, MgCO₃ serves a dual role as an internal pH buffer and a dynamic pore-forming agent, which effectively enhanced drug loading while ensuring smooth, attenuated initial burst release kinetics for over 18 days. In preclinical rodent models, weekly subcutaneous administration of Leo-micro@MD successfully eradicated EMS progression by downregulating TNF-α, which repaired aberrant estrogen receptor α (ERα) and progesterone receptor B (PRB) endocrine signaling while reactivating LC3B-mediated autophagy and Bcl-2-associated X protein/B-cell lymphoma 2 (Bax/Bcl-2)-dependent apoptosis in ectopic lesions. Meanwhile, Leo-micro@MD significantly suppressed synovial hyperplasia, restored bone integrity, and normalized articular matrix homeostasis in adjuvant-induced arthritis (AIA) rats as corroborated by comprehensive histopathological assessment and micro-CT imaging. Overall, this study introduces a translatable controlled-release strategy that expands the horizon of universal biomaterial platforms for the management of chronic inflammatory diseases.

**Scheme 1:**
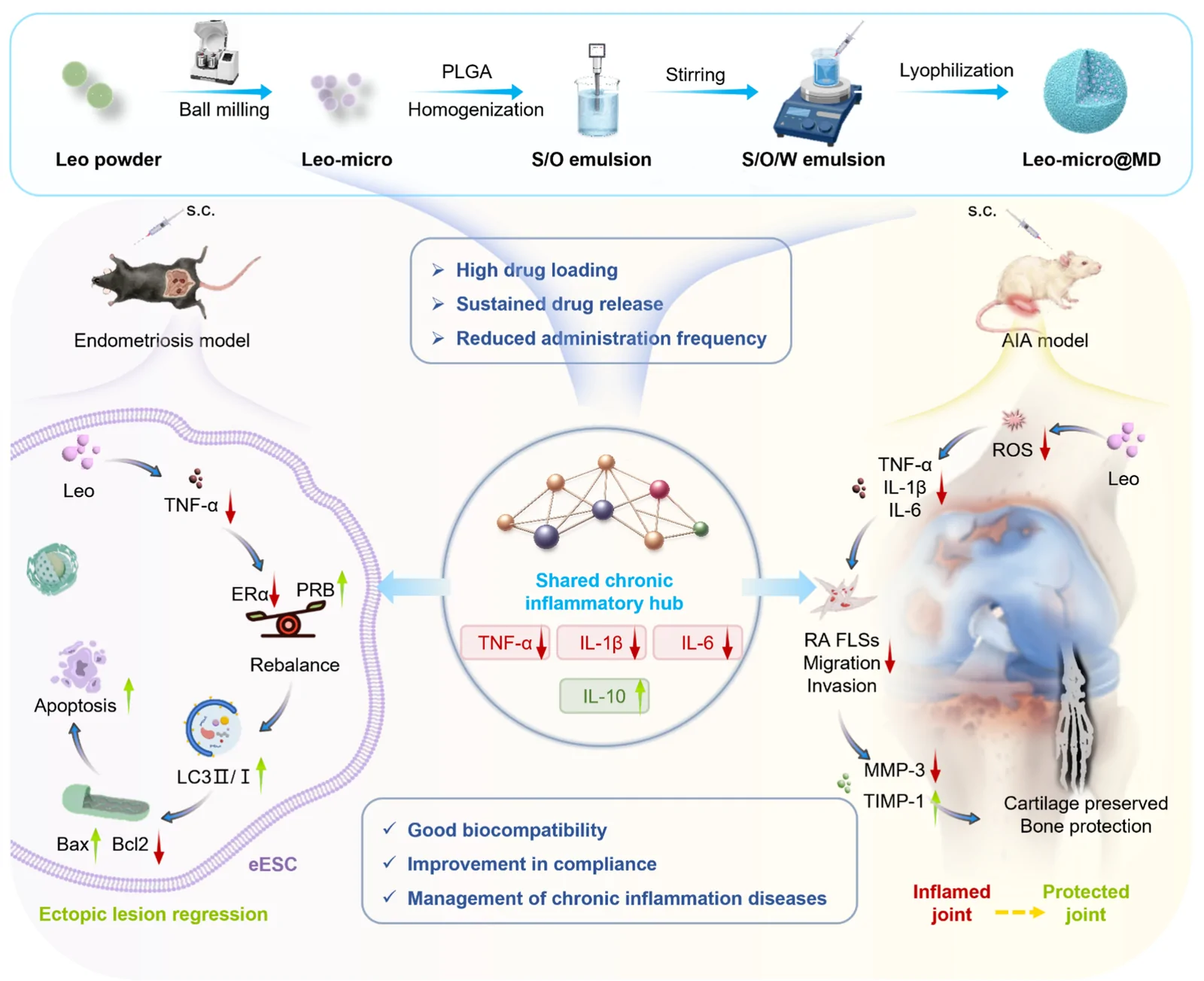
Schematic illustration of the preparation of Leo-micro@MD and their therapeutic effects against EMS and RA.

## 2 Experimental section

### 2.1 Materials

Leonurine (Leo) was obtained from Xinxiang Tianfeng Fine Chemical Co., Ltd. (Henan, China). Poly(lactide-*co-*glycolide) (PLGA) copolymers (Mw = 40 kDa, lactic (LA): glycolic acid (GA) = 75: 25; Mw = 75 kDa, LA: GA = 75: 25; Mw = 110 kDa, LA: GA = 75: 25; Mw = 75 kDa, LA: GA = 50: 50; Mw = 49 kDa, LA: GA = 50: 50, carboxyl-terminated; Mw = 75 kDa, LA: GA = 75: 25, ester-terminated) were supplied by Daigang Biology Co., Ltd. (Jinan, China). MgCO_3_, NaCl, and Dichloromethane were purchased from Macklin Co., Ltd. (Shanghai, China). Poly(vinyl alcohol) 0588 (PVA-205) was obtained from Aldrich Chemical (HE, Germany). Resazurin, Mineral oil, and lipopolysaccharide (LPS) were obtained from Sigma-Aldrich Inc. (MO, USA). The mouse and rat TNF-α ELISA kits, IL-1β ELISA kits, IL-6 ELISA kits, and IL-10 ELISA kits were obtained from Multi-Science Co., Ltd. (Shanghai, China). Estradiol ELISA was purchased from Demeditec (Germany). Crystal Violet Staining Solution, BCA protein assay kit, Autophagy Staining Assay Kit with MDC, and Terminal deoxynucleotidyl transferase-mediated nick end labeling (TUNEL) kit were purchased from Beyotime Biotechnology Co., Ltd. (Shanghai, China). 4,6-diamidino-2-phenylindole (DAPI) was purchased from Thermo Fisher Scientific (MA, USA). Anti-Progesterone Receptor (PR) antibody, anti-Estrogen Related Receptor (ER) antibody, and anti-Bax antibody were purchased from Hangzhou HuaAn Biotechnology Co., Ltd. (Hangzhou, China). Anti-GAPDH antibody was purchased from Santa Cruz Biotechnology (CA, USA). Anti-Bcl2 polyclonal antibody was purchased from Proteintech (IL, USA). Anti-LC3 was purchased from Medical & Biological Laboratories Co., Ltd. (Tokyo, Japan). Anti-TNF-α polyclonal antibody was purchased from ServiceBio Technology Company (Wuhan, China). The aspartate transaminase (AST) kit, alanine aminotransferase (ALT) kit, blood urea nitrogen (BUN) kit, and creatinine (CRE) kit were obtained from Nanjing Jiancheng Bioengineering Institute (Jiangsu, China). *Mycobacterium tuberculosis* was obtained from Becton, Dickinson, and Company (NJ, USA). Type IV Collagenase was purchased from Mu Biotech (Guangzhou, China). Dulbecco's Modified Eagle Medium/Nutrient Mixture F12 Ham (DMEM/F12), Dulbecco's Modified Eagle medium (DMEM), fetal bovine serum (FBS), penicillin-streptomycin stock solutions (PS), and trypsin-EDTA (0.25%) (Catalog No. 25200–072) were obtained from GIBCO BRL (NY, USA).

Animal experiments were reported in accordance with the ARRIVE 2.0 guidelines. The female C57BL/6 mice (6–8 weeks) and the male Sprague-Dawley rats (SD rats, 160–220 g) were provided by Zhuhai Best BioTech Co., Ltd. (Guangdong, China). They were housed in a clean room, with access to water *ad libitum*, a 12:12 h light-dark cycle (7:00 am - 7:00 pm), and a temperature of 25 ± 1 °C. All animal experimental protocols were performed in accordance with the Guidelines for Care and Use of Laboratory Animals of Macau University of Science and Technology and approved by the Animal Ethics Committee of Macau University of Science and Technology (MUST-NSFC-20230224001). For all therapeutic experiments, the individual animal was considered the experimental unit. Animals were allocated to the indicated experimental groups after model establishment using a random-number sequence. Each treatment group contained six animals (*n* = 6). The sample size was determined based on previous studies using the same disease models while minimizing animal use in accordance with the 3Rs principle. Where feasible, outcome assessment and quantitative analysis, including histological image analysis, micro-CT quantification, and biochemical measurements, were performed using coded samples by investigators blinded to treatment allocation. No animals or data points were excluded from the final analyses, and all animals allocated to each experimental group completed the study and were included in the statistical analysis.

Ectopic endometrial stromal cells (eESCs) were extracted from the ectopic lesion of mice with successfully established EMS models, and endometrial stromal cells (nESCs) were extracted from the endometrium of healthy mice. The eESCs and nESCs were cultured in DMEM/F12 containing 10% FBS and 1% PS. MH7A cells (the human rheumatoid arthritis synovial fibroblast cell line) and RAW264.7 cells (mouse monocyte macrophage) were obtained from the Cell Bank of the Chinese Academy of Sciences (Shanghai, China). They were cultured in DMEM containing 10% FBS and 1% PS. All the cells were cultured at 37 °C in a cell incubator with 5% CO_2_.

### 2.2 Preparation of Leo-micro@MD

Micronized Leo (Leo-micro) was prepared by planetary ball milling according to previous reports. More specifically, 250 mg of Leo powder and 2 mL Milli-Q water were added to the planetary ball mill (PM 100 CM, Retsch, Germany), and ground with 30 g zirconium beads (d = 1.2 mm) at 500 rpm for 10 min. Subsequently, the ground suspension was collected and lyophilized to obtain Leo-micro.

Leo-micro@MD was prepared by the method mentioned in the previous paper, which was the solid-in-oil-in-water (S/O/W) emulsion-solvent evaporation method. To be specific, 225 mg PLGA was dissolved in 3 mL methylene chloride, and Leo-micro in different mass ratios (15%, 25%, 35%) and MgCO_3_ in different mass ratios (0%, 1%, 3%) were added to the PLGA solution. The mixture was homogenized at 10,000 rpm for 1 min to obtain the S/O emulsion. It was poured into a 40 mL 5% PVA solution containing 5% sodium chloride (ice bath or room temperature), and then it was stirred at 600 rpm for 1 h. Next, the formed S/O/W emulsion was poured into 400 mL 0.5% PVA solution, magnetically stirred at 600 rpm, and the solvent was evaporated for 4 h. Finally, the microspheres were washed with Milli-Q water three times during filtration and lyophilized with a freeze dryer (Labconco, America). Leo-micro@MD was stored at −20 °C before use.

### 2.3 Characterization of Leo-micro@MD

#### 2.3.1 Drug loading and encapsulation efficiency

5 mg Leo-micro@MD was accurately weighed and dissolved in 1 mL acetonitrile. When it was completely dissolved, 9.0 mL of 0.1% formic acid aqueous solution was added and filtered through a 0.45 μm water filtration membrane. The content of Leo was quantified at 277 nm using UV spectrophotometry. The drug loading (DL) and encapsulation efficacy (EE) were calculated as follows.

$$\text{Drug Loading}\;\left(\mathrm{DL}\right)=\frac{\text{Weight of}\;\mathrm{Leo}\;\text{in}\;\mathrm{Leo}-\text{micro}@\mathrm{MD}}{\text{Weight of}\;\mathrm{Leo}-\text{micro}@\mathrm{MD}}\times 100\%$$

$$\text{Encapsulation Efficacy}\;\left(\mathrm{EE}\right)=\frac{\text{Weight of the actual}\;\mathrm{Leo}\;\text{loading}}{\text{Weight of theoretical}\;\mathrm{Leo}\;\text{loading}}\times 100\%$$

#### 2.3.2 Drug release behavior *in vitro*

20 mg Leo-micro@MD was placed into a dialysis bag (Molecular weight cut-off: 8000–14,000), and 1 mL PBS (pH = 7.4) was added; then the dialysis bag was placed in 20 mL PBS (pH = 7.4) and stirred at 100 rpm and 37 °C. At the predetermined time points, 2 mL of medium was collected and the same volume of fresh buffer was replaced. The concentration of Leo was determined by UV spectrophotometry at 277 nm, and the cumulative release rate was calculated using the following formula. The *in vitro* release data of Leo-micro@MD were fitted to zero-order, first-order, Higuchi, and Weibull kinetic models, and model performance was evaluated using R^2^ and Akaike information criterion (AIC). The Korsmeyer-Peppas model was additionally applied to the initial portion of the release profile (≤ 60% cumulative drug release) to calculate the release exponent (n) and characterize the predominant release mechanism.

$$\text{Cumulative release rate}\;\left(\%\right)=\frac{M_{t}}{M_{0}}\times 100\%$$

where M_t_ is the amount of drug released at time t, and M_0_ is the total amount of drug loaded in Leo-micro@MD.

#### 2.3.3 Scanning electron microscopy (SEM) and particle size analysis

The morphology of the microspheres was observed by scanning electron microscopy (SEM, Apreo 2S HiVac, Thermoscientific, USA). The microspheres were sputter-coated with gold and were scanned and photographed under the microscope at 10.0 kV acceleration voltage. The particle size distribution of Leo-micro@MD was quantified by the ImageJ software (National Institutes of Health, Bethesda, MA, USA) from three SEM images for 100 particle diameters. To further characterize the dispersion behavior of Leo-micro in the aqueous phase, its hydrodynamic particle size and zeta potential were measured by dynamic light scattering (DLS, Nano ZS90, Malvern, UK) using a particle-size analyzer. Leo-micro was dispersed in ultrapure water at an appropriate concentration, gently homogenized to obtain a uniform suspension, and immediately subjected to measurement. Each sample was measured in triplicate.

#### 2.3.4 Fourier transform infrared (FT-IR) spectroscopy

The structure of Leo in Leo-micro@MD was analyzed by a Fourier transform infrared spectrometer. Leo, Leo-micro, PLGA, the physical mixture of Leo-micro and PLGA, and Leo-micro@MD were mixed with potassium bromide, compressed into disks by a hydraulic press, and scanned with the Fourier transform infrared spectrometer (FT-IR, Iraffinity-1 s, Shimadzu, Japan) from 4000 to 500 cm^−1^. The spectral recording range was 400–4000 cm^−1^ with a resolution of 4 cm^−1^.

#### 2.3.5 X-ray diffraction (XRD)

The physical solid states of Leo, Leo-micro, PLGA, the physical mixture of Leo-micro and PLGA, and Leo-micro@MD were examined by X-ray powder diffraction (XRD, D8 Advance, Bruker, Germany). The XRD patterns were analyzed with an X'Pert PRO MPD at a scan rate of 2.07° min^−1^ and in the 2θ range of 5°–60°.

#### 2.3.6 Differential scanning calorimetry (DSC)

The thermal properties of drugs in microspheres were evaluated by Differential Scanning Calorimetry (DSC, TA DSC2500, Waters, USA). Leo, Leo-micro, PLGA, the physical mixture of Leo-micro and PLGA, and Leo-micro@MD were placed in the Tzero aluminum pan and heated from 10 °C to 300 °C at a scan rate of 10 °C min^−1^.

#### 2.3.7 Degradation behaviors

To evaluate the degradation ability of microspheres, 100 mg each of blank microspheres, blank microspheres (3% MgCO_3_) and Leo-micro@MD(3% MgCO_3_) were accurately weighed and added to 10 mL PBS (pH = 7.4), respectively. At predetermined time points, the microspheres were collected and washed with Milli-Q water. After lyophilization, the microspheres were weighed, and the degradation rate was calculated with the following formula:

$$\text{Weight loss}\;\left(\%\right)=\frac{M_{0}-M_{t}}{M_{0}}\times 100\%$$

where M_t_ is the weight of the remaining microsphere at time t, and M_0_ is the initial weight of the microsphere.

### 2.4 Therapeutic effects of Leo-micro@MD on EMS *in vitro*

#### 2.4.1 Establishment of the EMS model and extraction of the primary cells

The female C57BL/6 mice (6–8 weeks) were provided by Zhuhai Best BioTech Co., Ltd. (Guangdong, China). They were housed in a clean room, with access to water *ad libitum*, a 12:12 h light-dark cycle (7:00 am - 7:00 pm), and a temperature of 25 ± 1 °C. All animal experimental protocols were performed in accordance with the Guidelines for Care and Use of Laboratory Animals of Macau University of Science and Technology and approved by the Animal Ethics Committee of Macau University of Science and Technology.

The endometriosis (EMS) model was established with C57BL/6 mice aged 6–8 weeks. After 2 weeks of adaptive feeding, estradiol (E2) was injected intraperitoneally three times a week (0.2 μg/g body weight), as previously reported. According to the results of the vaginal smear, mice in estrus were selected as recipients for the EMS model. A donor mouse was euthanized and the uterus was removed. The endometrium was scraped and cut into fragments about 1 mm in diameter, which were then injected into the abdominal cavity of the two recipient mice. Each recipient mouse was then intraperitoneally injected with estradiol twice within a week to improve the success rate of modeling. The mice were fed under normal conditions for 2 weeks for follow-up experiments.

Ectopic endometrial stromal cells (eESCs) were extracted from mice with a successfully established EMS model, and endometrial stromal cells (nESCs) were extracted from the mouse endometrium. Specifically, the endometrial or ectopic endometrial lesion tissue was broken into 1–2 mm^3^ fragments, then added to DMEM/F12 medium containing 1 mg/mL type IV collagenase and digested for 20 min at 37 °C. The dissociated cells were followed by filtration with a 400-mesh silkscreen, while larger tissue blocks were subjected to the above digestion and filtration. The digested and filtered cells were collected and centrifuged at 1500 rpm for 10 min. Then, the supernatant was discarded and fresh medium was added to resuspend the cells. The suspended cells were centrifuged at 1200 rpm for 5 min and the supernatant was discarded to remove the red blood cells. Fresh DMEM/F12 media with 10% FBS were added again. The cells were incubated overnight in the cell incubator at 37 °C, 5% CO2, then washed with PBS to remove the suspended cells, and the adherent stromal cells were further cultured for subsequent experiments.

#### 2.4.2 Cell viability assessment

To evaluate the cytocompatibility of Leo-micro@MD, an indirect Transwell-based exposure system was employed. Given the microscale diameter of Leo-micro@MD and its intended function as an extracellular drug depot, the microspheres were placed in the upper chamber of a Transwell insert (pore size, 5 μm; Millipore, MA, USA), while cells were cultured in the lower chamber. This configuration minimized direct microsphere sedimentation and particle–cell contact while allowing released Leo to diffuse across the membrane and reach the cells (Liang et al., 2022). The freeze-dried Leo-micro@MD was sterilized by ultraviolet irradiation for 4 h. The diluted concentration of Leo-micro@MD was calculated according to the release curve *in vitro*. Leo-micro@MD at different concentrations (0, 10, 50, 100, 200, and 500 μg/mL) was placed in Transwell inserts and incubated with nESCs and eESCs for 24 h. Cell viability was subsequently evaluated using the Alamar Blue™ assay (Yang et al., 2026) (Wu et al., 2026). After co-incubation, the cells were treated with 10% Alamar Blue and incubated for another 4 h. The supernatant was collected, and the absorbance at 570 nm and 600 nm was measured using the microplate reader (SpectraMax ID5, Molecular Devices, USA). The cell viability was calculated according to the manufacturer's instructions.

#### 2.4.3 Invasion assay *in vitro*

The effect of Leo-micro@MD on the invasion ability of eESCs cells was investigated by a transwell-mediated invasion experiment. The transwell inserts with a pore size of 8 μm were coated with matrix gel and held at 37 °C for 1 h. The nESCs and eESCs were suspended in serum-free medium and inoculated into the insert, while the bottom chamber was filled with medium containing 10% fetal bovine serum. Free Leo and Leo-micro@MD (equivalent to 50 μm Leo) were added and incubated for 24 h. The cells were then fixed with 4% PFA and stained with 0.1% crystal violet. Then, cells inserted into the upper part of the membrane were removed with a cotton swab. The number of infiltrating cells in the three random fields was quantified under an optical microscope (CKX53, Olympus, Japan).

#### 2.4.4 Anti-inflammatory and anti-estrogen expression effects *in vitro*

nESCs and eESCs were seeded in 24-well plates, respectively, and then incubated with free Leo (50 μM) and Leo-micro@MD (placed in a transwell chamber with a concentration of 50 μM of Leo released 24 h) for 24 h. Subsequently, the supernatant of the cell cultures was collected and the levels of TNF-α, IL-1β, IL-6, IL-10, and estrogen were measured according to the ELISA kit instructions.

#### 2.4.5 Western blot

As previously described, free Leo and Leo-micro@MD (equivalent to a Leo dose of 60 μM) were incubated with eESCs in the transwell chambers for 48 h. Total proteins from eESCs were subsequently extracted. Protein quantification was performed using the BCA kit according to the instructions. The protein levels of estrogen receptor α (ERα), progesterone receptor subtype B (PRB), Bax, Bcl-2, and LC3BII/I were analyzed according to the standard procedure of western blot analysis.

#### 2.4.6 Autophagy assay by MDC kit

The effect of Leo-micro@MD on the autophagy of eESCs was studied by using an autophagy staining detection kit (MDC method). The eESCs were seeded on the glass coverslips of the 24-well plate, and then free Leo and Leo-micro@MD were placed in Transwell chambers and incubated with the cells for 24 h. The diluted MDC staining solution was added to the 24-well plate and co-incubated with cells in a dark incubator for 50 min according to the MDC kit instructions. After washing three times with assay buffer, the cells were fixed with 4% PFA, and the cell nucleus was stained with DAPI. The cell fluorescence signals were observed with a confocal laser scanning microscope (CLSM, Leica Stellaris, Germany). The fluorescence intensity was semi-quantified by ImageJ software, and the result was determined to be the average of three randomly selected microscope fields.

#### 2.4.7 Apoptosis assay by TUNEL kit

The transferase-mediated digoxigenin-dUTP nick-end labeling (TUNEL) assay was conducted using a one-step TUNEL cell apoptosis detection kit. Firstly, eESCs were inoculated into 24-well plates, and free Leo and Leo-micro@MD were incubated with cells in the transwell chamber for 24 h. After incubation, the cells were fixed with 4% PFA, washed with PBS, and permeabilized with 0.1% Triton X-100. The cells were then incubated with TUNEL staining mixture in 37 °C darkness for 1 h. Finally, the cells were washed with PBS and stained with DAPI. The cell fluorescence signals were observed with CLSM. The fluorescence intensity was semi-quantified by ImageJ software, and the result was determined to be the average of three randomly selected microscope fields.

### 2.5 Therapeutic Effects of Leo-micro@MD on RA *in vitro*

#### 2.5.1 Cell viability assessment

Similar to the description in part 2.4.2, the cell viability of Leo-micro@MD on MH7A cells was measured by the Alamar Blue™ test (Liu et al., 2025). In short, different concentrations of Leo-micro@MD (1–1000 μM) were placed in transwell chambers to co-incubate for 24 h with MH7A cells. After incubation with 10% Alamar Blue for another 4 h, the absorbance was measured at 570 nm and 600 nm. Cell viability was calculated by description.

#### 2.5.2 Anti-inflammatory effects *in vitro*

To investigate the anti-inflammatory activity of Leo-micro@MD, Raw264.7 cells were inoculated in 24-well plates overnight, and then Raw264.7 cells were activated with 1 μg/mL LPS for 6 h, except for the control group. The treatment groups were incubated with free Leo (50 μM) and Leo-micro@MD (placed in a transwell chamber with the concentration of 50 μM of Leo released for 24 h) for 24 h. Subsequently, the supernatant of the cell cultures was collected and the levels of TNF-α, IL-1β, IL-6, and IL-10 were measured according to the ELISA kit instructions.

#### 2.5.3 Migration assay and transwell invasion assay *in vitro*

The effect of Leo-micro@MD on the migration behavior of MH7A cells was studied by a wound healing experiment. To be specific, MH7A cells were inoculated into a 24-well plate and activated with LPS (1 μg/mL) for 12 h. When the cells reached 90–100% fusion, a straight line was scraped with the pipette tip, washed with PBS, and added to serum-free medium for culture. Then, free Leo and Leo-micro@MD (placed in a transwell chamber with a concentration of 60 μM of Leo released for 48 h) were incubated with the cells for 48 h. The microscope (CKX53, Olympus, Japan) was used to observe and shoot, and the relative migration area was calculated according to the following formula.

$$\text{Relative migration area}=\frac{A_{0\;h-}A_{48\;h}}{A_{0\;h}}\times 100\%$$

where A_0 h_ and A_48 h_ stand for the scratch area at 0 h and 48 h, respectively.

The effect of Leo-micro@MD on the invasion ability of MH7A cells was investigated by a transwell-mediated invasion experiment. MH7A cells were activated with 1 μg/mL LPS for 12 h, then transwell inserts (pore size 8 μm) were wrapped in matrix gel at 37 °C for 1 h. Subsequently, activated MH7A cells were collected, inoculated with serum-free medium in the inserts, and incubated with free Leo, Leo-micro@MD (equivalent to 60 μM Leo) for 24 h. The bottom chamber was filled with a medium containing 10% fetal bovine serum. The cells were then fixed with 4% PFA and stained with 0.1% crystal violet. Cells inserted into the upper part of the membrane were removed with a cotton swab. The number of invasive cells was quantified in three random fields under an optical microscope.

### 2.6 The therapeutic effect of Leo-micro@MD on EMS *in vivo*

#### 2.6.1 Evaluation of the therapeutic effect

The establishment of the EMS model was described in 2.4.1. On the first day of modeling, the mice were randomly divided into 4 groups (6 mice per group), which were as follows: (1) Control group: healthy mice; (2) Model group: model mice were injected subcutaneously with PBS every day; (3) Free Leo group: the modeling mice were injected subcutaneously with free Leo every day (10 mg/kg/ day, evenly dispersed into 0.5% *w*/*v* CMCNa); (4) Leo-micro@MD group: modeling mice were injected subcutaneously with Leo-micro@MD every 7 days. Briefly, Leo-micro@MD was uniformly dispersed in 0.5% (*w*/*v*) CMC-Na before administration and injected into the subcutaneous tissue of the axillary region. The injection volume was individually adjusted according to body weight and formulation concentration to achieve the prescribed Leo-equivalent dose. Free Leo was administered subcutaneously once daily at 10 mg/kg, whereas Leo-micro@MD was administered once every 7 days at 70 mg/kg Leo-equivalent per injection, corresponding to the cumulative 7-day dose of free Leo. All mice were weighed every 2 days. After 28 days of treatment, blood was extracted from the eyeballs of all mice, centrifuged at 3500 rpm for 10 min, and serum was collected. After euthanizing the mice, 2 mL of physiological saline was injected intraperitoneally, and then the abdomen was gently massaged to remove the peritoneal lavage solution. Mice with ectopic endometrial lesions were then collected, measured, and photographed. Finally, all the major organs of the mice were extracted, including the liver, heart, lung, spleen, and kidney, and the visceral index was calculated.

#### 2.6.2 TUNEL assay

On day 28, the ectopic lesion of each group was collected, fixed with 4% PFA, and then paraffin sections of the ectopic lesions were obtained. According to the manufacturer's instructions for the TUNEL staining kit, sections of ectopic lesions were treated with 20 μg/mL proteinase K at room temperature for 20 min and rinsed 3 times. TUNEL detection solution was then added to the lesion slices and incubated in the dark and damp room at 37 °C for 1 h. After three washes, the nuclei were stained with DAPI. The sections were observed by CLSM and fluorescence intensity was analyzed by ImageJ.

#### 2.6.3 H&E and Masson staining

The slices of EMS lesions were stained with H&E and Masson. The stained sections were observed and photographed under the microscope (DM2500, Leica Stellaris, Germany).

#### 2.6.4 Western blot

The protein was extracted from mouse ectopic lesions using RIPA buffer containing a protease inhibitor and quantified using the BCA protein detection kit. Subsequently, the expression of ERα, PRB, Bax, Bcl-2, and LC3BII/I protein levels were detected by Western blot. At the same time, the optical density of the bands was analyzed by ImageJ.

#### 2.6.5 Immunohistochemistry

On day 28, the ectopic lesions of each group were collected, fixed with 4% PFA, and then paraffin sections of the ectopic lesions were obtained. According to the manufacturer's instructions, lesion sections were immunostained with TNF-α, PRB, and ERα, and quantitative detection was performed by ImageJ to evaluate the expression level of related proteins in ectopic tissues.

#### 2.6.6 Peritoneal level of TNF-α, IL-1β, IL-6, IL-10, and Estradiol

The levels of TNF-α, IL-1β, IL-6, IL-10, and Estradiol in the collected peritoneal lavage fluid were quantified by ELISA according to the instructions provided by the assay kits.

#### 2.6.7 Safety evaluation

On day 28, serum and the major organs (heart, liver, spleen, lung, and kidney) of mice in each group were collected, and ALT, AST, CRE, and BUN levels in the serum were determined by commercial kits according to the manufacturer's instructions. Besides, H&E staining of the major organs was performed to analyze the potential toxicity.

### 2.7 Pharmacokinetic study

The SD rats were randomly divided into 2 groups, with 6 rats in each group. The groups were the Free Leo group (10 mg/kg) and the Leo-micro@MD group (70 mg/kg of Leo dose), and a single subcutaneous injection of Leo-micro@MD was administered. Blood samples were collected from the orbital venous plexus at predetermined time points after administration. After each blood collection, an equal volume of sterile normal saline was administered when necessary to compensate for blood-volume loss. Blood samples were centrifuged at 3500 rpm at 4 °C for 15 min, and the separated plasma was collected and stored at −80 °C until analysis.

Plasma leonurine concentrations were quantified using a validated liquid chromatography–tandem mass spectrometry (LC–MS/MS, Agilent-6460, LC-QQQ, Agilent, USA) method. Briefly, plasma samples were processed by protein precipitation, with Nα-Benzoyl-L-Arginine Ethyl Ester Hydrochl (BAEE) used as the internal standard. Chromatographic separation was performed on a C18 column (Agilent Zorbax SB-C18, 150 × 2.1 mm, 5 μm) maintained at 27 °C. The mobile phase consisted of acetonitrile: 10 mM ammonium acetate buffer (pH = 4.0) 10: 90 (*v*/v) delivered at a flow rate of 0.3 mL/min under elution conditions. Quantification was performed in multiple-reaction monitoring (MRM) mode using the precursor-to-product ion transition of *m*/*z* 312.2 → 180.8 for leonurine and *m*/*z* 307.3 → 104.6 for the internal standard. The ion-source voltage and temperature were set at 5500 V and 400 °C, respectively, with nitrogen used as the drying gas. The entrance and exit potentials were 10 and 8 V, respectively. The declustering potential and collision energy were 76 and 26 V for Leo and 68 and 32 V for BAEE, respectively. The calibration curve was linear over the range of 1–1000 ng/mL, with correlation coefficients of >0.999. The intra- and inter-day precision evaluated at 5, 50, and 500 ng/mL showed RSD values below 5.5%, while the corresponding accuracies ranged from 86.92% to 104.26%. Method and extraction recoveries ranged from 91.29% to 104.79% and from 93.22% to 104.10%, respectively, demonstrating satisfactory analytical performance for the determination of Leo in rat plasma. Pharmacokinetic parameters were calculated from the plasma concentration–time profiles by noncompartmental analysis using Phoenix WinNonlin 8.3.5. Numerical deconvolution was additionally performed using Phoenix WinNonlin 8.3.5 to characterize the temporal *in vivo* input profile of Leo-micro@MD.

### 2.8 The therapeutic effect of Leo-micro@MD on RA *in vivo*

#### 2.8.1 Establishment of adjuvant-induced arthritic (AIA) rat model

The rat model of adjuvant-induced arthritis (AIA) was the same as previously reported (Xiao et al., 2026). In short, mineral oil was ground to emulsify heat-inactivated *Mycobacterium tuberculosis*, and then the AIA model was induced by subcutaneous injection of the suspension agent (100 μL) into the rat tail root.

#### 2.8.2 Treatment of AIA rats

24 SD rats were randomly divided into 4 groups with 6 rats in each group: (1) Control group (healthy rats); (2) Model group (AIA rats injected subcutaneously with equal volume of PBS every day); (3) Free Leo group (AIA rats received subcutaneous injection of 10 mg/kg/ day of Leo) and (4) Leo-micro@MD group (AIA rats received subcutaneous injection of Leo-micro@MD every 7 days at a dose of 10 mg/kg/ day of Leo). The body weight, arthritis score, and hind paw volume of each group were monitored every 3 days for 30 consecutive days.

In addition, arthritis scores were recorded according to previously reported clinical symptom assessment methods. Each paw was scored on a scale of 0–4, 0 = no swelling or erythema, 1 = mild swelling or erythema, mild redness of ankle or tarsal joint, 2 = low-moderate edema, mild redness of ankle or tarsal joint, 3 = significant edema, limited use of joint, moderate redness of ankle to metatarsal joint, 4 = severe edema, stiffness of joint, Severe redness of ankles and feet. All rats were euthanized on day 30 of treatment, and blood, major organs, and hind paws were collected for further investigation.

#### 2.8.3 Levels of inflammatory cytokines

The blood of each group was centrifuged at 2000 rpm for 10 min, and serum was collected. Serum levels of inflammatory cytokines TNF-α, IL-1β, IL-6, IL-10, matrix metalloproteinase MMP-3, and TIMP-1 were measured by an ELISA kit.

#### 2.8.4 Micro-computed tomography (micro-CT) analysis

Dissected ankle joints were evaluated by the micro-CT scanner (SkyScan1176, Bruker, Belgium) with the following parameters: current 385 μA, voltage 85 kV, exposure time 65 ms, resolution 35 μm, and aluminum filter 1 mm. The 3D visual images were reconstructed using CT-Vol software (Bruker-micro-CT, Belgium), and the photos were reconstructed using NRecon software. To obtain quantitative results for different groups, the morphological parameters of trabeculae bone were quantified using the CTAn procedure (Skyscan, Bruker, Belgium), such as bone mean mineral density (BMD), bone volume fraction (BV/TV, %), tissue mineral density (TMD), total porosity, trabecular number (Tb. N, 1 mm^−1^), trabecular bone spacing (Tb. Sp, μm), trabecular thickness (Tb. Th, μm) and structural model index (SMI) of the tibia.

#### 2.8.5 Histopathology of inflamed joints

Dissected ankle joints in each group were fixed in 4% PFA, decalcified, and then embedded in paraffin blocks and cut into 5 μm thick slices, stained with hematoxylin and eosin (H&E), saffron O-fast green (SOFG) and Masson's trichrome (Masson), respectively. The stained sections were observed under a microscope and photographed.

#### 2.8.6 Safety evaluation

The serum levels of ALT, AST, CRE, and BUN were detected by a commercial kit according to the manufacturer's instructions. Major organs (heart, liver, spleen, lung, kidney) were stained with H&E, and their physiological structures were observed under the microscope.

### 2.9 Statistical analysis

All data were presented as mean ± standard deviation (SD). OriginPro 9.0 software (OriginLab, USA) was adopted for data processing and analysis. For *in vitro* experiments, *n* = 3 represents three independent experiments performed separately under the same experimental conditions. For *in vivo* experiments, each individual animal was considered one independent experimental unit, with *n* = 6 animals per group. Data distribution and homogeneity of variance were evaluated before parametric analysis using the Shapiro-Wilk test and Levene's test, respectively. Comparisons between two independent groups were performed using a two-tailed unpaired Student's *t*-test when the assumptions for parametric analysis were satisfied. Comparisons among three or more independent groups were analyzed by one-way analysis of variance (ANOVA) followed by Tukey's multiple-comparisons test. For longitudinal measurements obtained repeatedly from the same animals, including body weight and hind-paw volume, two-way repeated-measures ANOVA was used to evaluate the effects of treatment, time, and treatment and time interaction, followed by appropriate multiple-comparisons testing when required. Statistical significance was defined as *p* < 0.05.

## 3 Results and discussions

### 3.1 Preparation and characterization of Leo-micro@MD

To maximize the encapsulation efficiency (EE) and drug loading (DL) capacity of the delivery platform, the raw powders of Leo were initially micronized *via* high-energy planetary ball milling and systematically characterized (Xu et al., 2025). This milling process markedly reduced the particle size of raw Leo, thereby increasing the accessible surface area for polymer encapsulation and enhancing its potential to achieve high DL and EE (Yang et al., 2024). Scanning electron microscopy (SEM) revealed that Leo exhibited irregular crystalline structures with particle sizes ranging from 20 to 200 μm (Fig. 1a). After planetary ball milling, micronized Leo (Leo-micro) showed a substantially reduced and more homogeneous particle size, with an average size of approximately 2.36 ± 6.33 μm (Fig. 1b). Further analysis of the scattered light revealed that the particle size of Leo-micro was approximately 2.15 ± 1.35 μm, and the Zeta potential was −10.08 ± 2.45 mV, which was consistent with the quantitative results obtained by SEM (Fig. S1). The structural changes induced by milling were further examined by X-ray diffraction (XRD) and Fourier-transform infrared (FT-IR) spectroscopy. The broadened XRD peaks indicated a reduction in crystal-domain size and partial loss of crystallinity after milling (Fig. S1a). FT-IR spectra showed no appreciable shifts in the characteristic carbonyl stretching vibration (C=O, ~1680 cm-1) or amine stretching vibration (N—H, ~3348 cm-1), demonstrating that the chemical structure of Leo was preserved during the 10-min milling process (Fig. S1b). After ball milling, there was no significant difference in the solubility of Leo-micro and Leo in water and PBS (pH 7.4) (Fig. S3). HPLC analysis showed comparable chromatographic purity before and after ball milling, indicating that the micronization process did not cause detectable chemical degradation of Leo under the tested conditions (Fig. S4).

**Fig. 1:**
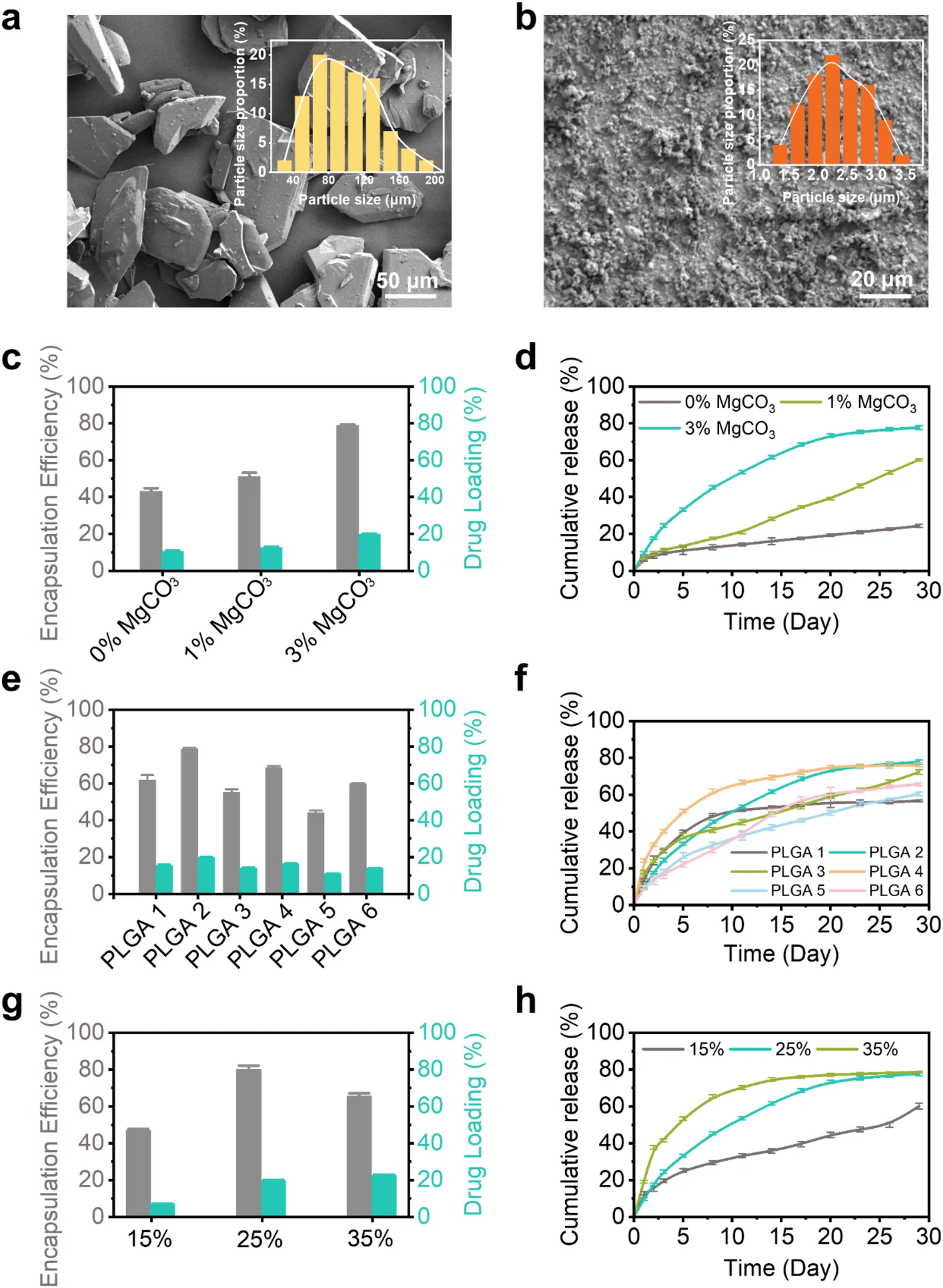
Preparation of Leo-loaded microcomposite depot. (a) Representative SEM images of Leo powder (scale bar = 50 μm) and the size distribution. (b) Representative SEM images of Leo-micro (scale bar = 20 μm) and size distribution quantified by ImageJ. The scale bars were specified on the images, respectively. Impact of additives on (c) encapsulation efficiency and drug loading capability and (d) drug release kinetics. Impact of PLGA type on (e) encapsulation efficiency, drug loading capability and (f) drug release kinetics. PLGA 1: 75/25-COOH (40 kDa), PLGA 2: 75/25-COOH (75 kDa), PLGA 3: 75/25-COOH (110 kDa), PLGA 4: 50/50-COOH (75 kDa), PLGA 5: 85/15-COOH (75 kDa), PLGA 6: 75/15-COOR (75 kDa). Impact of the initial drug loading dosage ratio on (g) drug loading capability and (h) drug release behavior. Data were presented as mean ± SD, *n* = 3.

After confirming the successful micronization of Leo, the formulation of Leo-loaded PLGA microcomposite depots (Leo-micro@MD) was systematically optimized to achieve high DL efficiency and controlled drug release. Emulsion-solvent evaporation is a widely used strategy for preparing PLGA microspheres (Clark et al., 2022). Nevertheless, conventional polymeric microspheres often suffer from intrinsic limitations, including pronounced burst release, insufficient drug encapsulation, and poorly regulated release kinetics, which collectively compromise their translational potential. In PLGA-based systems, drug release is primarily governed by the diffusion of the payload through the polymer matrix and the hydrolytic erosion of the polymer network (Martinez-Borrajo et al., 2023). Therefore, rational modulation of polymer properties and formulation parameters is essential for balancing DL, EE, and sustained-release performance. In this study, Leo-micro@MD were fabricated using a solid-in-oil-in-water (S/O/W) double emulsion-solvent evaporation method. Key formulation and processing variables, including additive concentration, PLGA molecular weight, terminal functional groups, osmotic pressure, preparation temperature, polymer concentration, and initial theoretical drug feeding ratio, were screened through single-factor experiments. Besides, MgCO_3_ was then introduced as a buffering and pore-forming excipient to regulate the internal microenvironment and release behavior of Leo-micro@MD. During PLGA degradation, acidic degradation products may accumulate within the microspheres, potentially affecting drug stability and release kinetics. The incorporation of MgCO_3_ can partially neutralize this acidic microenvironment while simultaneously modulating the internal pore architecture of the PLGA matrix (Clark et al., 2022). As shown in Fig. 1c and d, increasing the MgCO_3_ content enhanced the loading of Leo-micro and accelerated cumulative drug release. This effect may be attributed to two complementary mechanisms. First, the alkaline nature of MgCO_3_ elevated the internal pH during formulation fabrication, thereby reducing the protonation and aqueous solubility of the basic drug Leo-micro and limiting its leakage into the external aqueous phase. Second, the dissolution of MgCO_3_ generated interconnected pore channels within the PLGA matrix, facilitating water penetration and drug diffusion. Among the tested formulations, 3% MgCO_3_ achieved the optimal balance between high drug loading and controlled sustained release, while avoiding excessive porosity and premature drug leakage.

Subsequently, the impact of PLGA types was investigated, as the lactide-to-glycolide ratio, molecular weight, and terminal groups of PLGA directly affect polymer hydrophobicity, water uptake, matrix degradation, and drug release behaviors. As shown in Fig. 1e, microcomposite depots prepared with PLGA (75/25-COOH, 75 kDa) exhibited the highest DL and EE among the tested formulations. In addition, formulations prepared with higher-molecular-weight and ester-terminated PLGA displayed delayed drug release, which was consistent with the slower hydration and degradation of these PLGA matrices (Fig. 1f). These results indicate that PLGA (75/25-COOH, 75 kDa) provided a favorable balance between efficient Leo encapsulation and sustained drug release, and was therefore selected as the polymer matrix for subsequent formulation optimization (Song et al., 2023).

Furthermore, the external osmotic pressure, preparation temperature, and theoretical drug feeding ratio were also optimized to refine microsphere structure and drug-release performance. To regulate the osmotic gradient between the internal and external phases, sodium chloride was added to the outer aqueous phase. Adjustment of the external osmotic pressure can alter water transport and drug diffusion during formation, thereby influencing matrix density and encapsulation efficiency. As shown in Fig. S5, the addition of 5% sodium chloride significantly improved both DL and EE and promoted more complete cumulative drug release, suggesting that this osmotic condition favored the formation of structurally stable Leo-micro@MD. Preparation temperature affects solvent extraction, polymer solidification, and drug redistribution during formulation formation. As shown in Fig. S6, preparation under an ice-bath condition at 0 °C increased both DL and EE while suppressing the initial rapid release. This improvement was likely due to reduced drug diffusion into the external aqueous phase and the formation of a more compact microsphere shell at low temperature. Finally, the theoretical drug feeding ratio was evaluated to maximize drug loading without compromising microsphere integrity. As shown in Fig. 1g and h, increasing the initial drug loading dosage ratio from 15% to 25% increased DL from 7.13 ± 0.18% to 11.43 ± 0.40%. However, when the feeding ratio was further increased to 35%, DL decreased, and drug release became excessively rapid. This phenomenon may be explained by the excessive amount of Leo-micro exceeding the loading capacity of the PLGA matrix, thereby disrupting microsphere integrity and creating additional diffusion pathways for rapid drug leakage.

Conclusively, through the systematic optimization of the aforementioned manufacturing parameters, the definitive formulation for the long-acting Leo-micro@MD platform was successfully established. Based on the screening results, carboxyl-terminated PLGA (75/25-COOH, 75 kDa) was selected as the optimal polymer matrix. The final processing regime was then established by integrating 3% MgCO₃ into the inner phase and 5% sodium chloride into the external phase, with emulsification performed at a solidification temperature of 0 °C and an initial theoretical drug feeding ratio of 25%.

Subsequent to optimizing the structural composition of the microcarriers, the morphology, particle size distribution, and solid-state characterization of Leo-micro@MD were systematically investigated. The SEM captured that the engineered Leo-micro@MD possessed a highly defined spherical geometry and a particle size comparable to that of blank microspheres, while uniquely manifesting a well-distributed porous surface architecture (Fig. 2a). Quantitative ImageJ analysis verified that both blank microspheres and Leo-micro@MD exhibited an average hydrodynamic diameter centered at approximately 45 μm (Fig. 2b and c). To elucidate the chemical structural integrity of the encapsulated components, FT-IR spectroscopy was performed. As shown in Fig. 2d, the characteristic absorption peaks of Leo-micro appeared at 3348 cm^−1^ (N—H) and 1680 cm^−1^ (C=O), while those of PLGA appeared at 1750 cm^−1^ (C=O) and 1150 cm^−1^ (C-O-C). Crucially, these diagnostic bands remained perfectly preserved in the composite Leo-micro@MD spectrum without broadening or frequency shifts, confirming the absence of adverse secondary physicochemical interactions between the PLGA matrix and the encapsulated Leo-micro during emulsification. Furthermore, the sharp diffraction peaks of Leo (at 21.94°, 24.14°, and 26.76°) remained distinct in the XRD spectrum of Leo-micro@MD, confirming that the drug was successfully encapsulated within the microspheres in its pristine crystalline form without undergoing any polymorphic transitions during preparation (Fig. 2e). DSC thermograms (Fig. 2f) revealed the glass transition temperature of PLGA at ~50 °C and the characteristic endothermic melting peak of Leo-micro at ~150 °C; notably, both peaks were preserved in the Leo-micro@MD profile, further confirming that the encapsulated drug retained its crystalline properties.

**Fig. 2:**
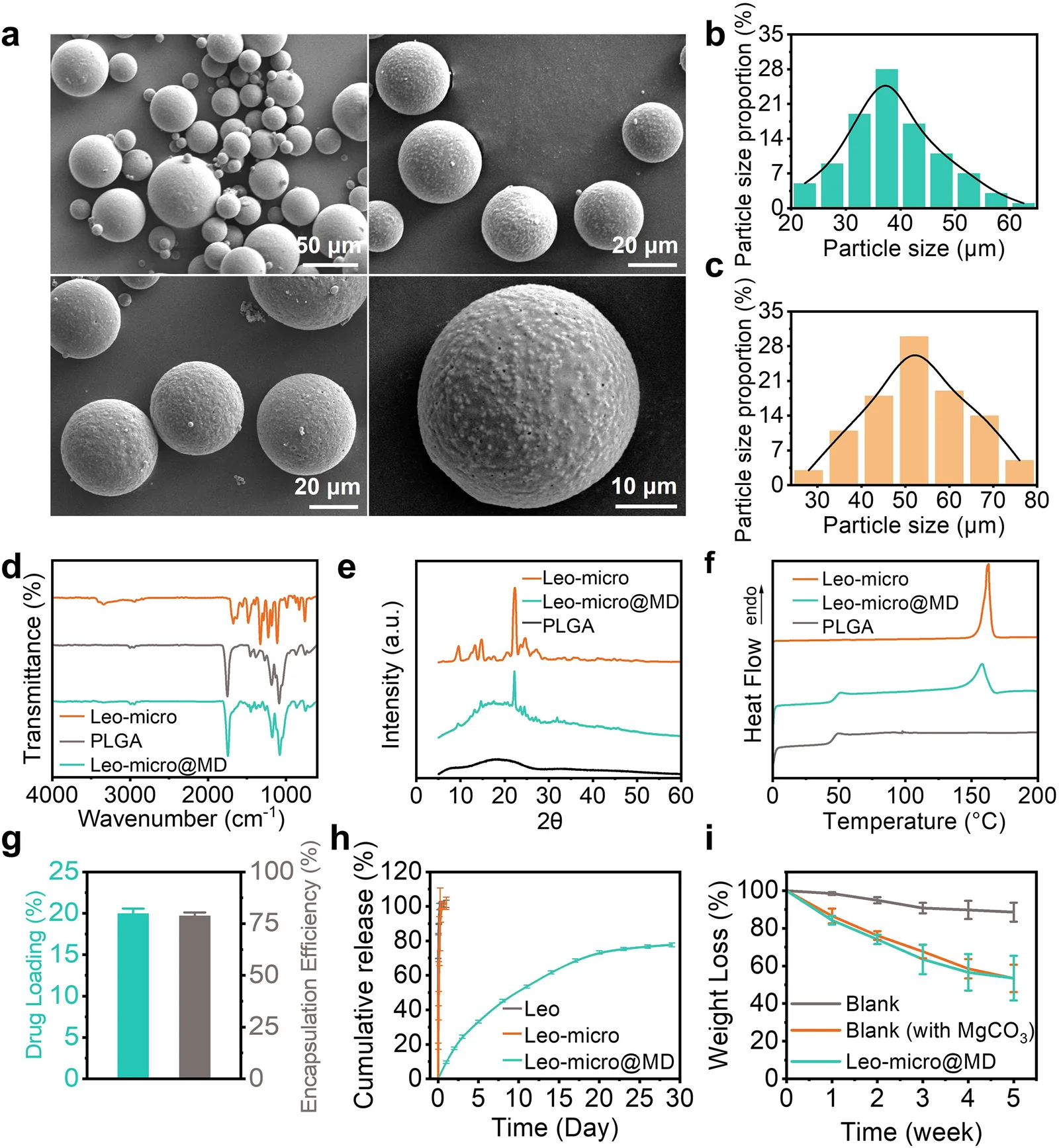
Characterization of Leo-micro@MD. (a) Representative SEM images of blank microspheres and Leo-micro@MD. The scale bars were specified on the images, respectively. Size distribution of (b) MP and (c) Leo-micro@MD. (d) FTIR spectra, (e) X-ray diffraction patterns, (f) DSC thermograms of Leo-micro, PLGA, and Leo-micro@MD. (g) Drug loading efficiency and encapsulation efficiency of Leo-micro@MD. (h) Drug release kinetics of Leo, Leo-micro, and Leo-micro@MD. (i) Weight loss of blank polymer microspheres (Blank), blank microspheres with 3% MgCO_3_ (Blank (with MgCO_3_)), and Leo-micro@MD after immersion in PBS at 37 °C for 5 weeks. Data were presented as mean ± SD, *n* = 3.

Moreover, the *in vitro* drug release kinetics and the underlying matrix hydrolytic degradation profiles of the optimized platform were comprehensively evaluated. Under the optimized preparation conditions, Leo-micro@MD achieved an outstanding EE of 78.8 ± 1.6% and DL of 19.9 ± 0.6% (Fig. 2g). In contrast to free Leo and Leo-micro, Leo-micro@MD exhibited a continuous and well-regulated release profile over 18 days without an obvious burst-release phase (Fig. 2h). Quantitative model fitting strengthened interpretation of this profile (Table S1). Among the models applied to the complete release dataset, the Weibull model provided the best overall description R^2^ approximately 0.993, and Akaike information criterion (AIC) approximately −184.6, followed closely by the first-order model. Fitting the initial ≤60% release phase to the Korsmeyer-Peppas equation yielded n approximately 0.77. For a spherical matrix, this value lies within the anomalous/non-Fickian range, indicating that Leo release is better described by coupled diffusion and matrix-mediated processes than by simple Fickian diffusion or ideal zero-order release alone. This sustained-release behavior indicated that PLGA microsphere encapsulation effectively converted the rapidly releasing Leo into a long-acting depot formulation, which might support prolonged drug exposure after administration. Although PBS (pH 7.4) provided a standardized and reproducible condition for characterizing the baseline sustained-release behavior of Leo-micro@MD, it does not fully reproduce the complex subcutaneous microenvironment; therefore, future studies will further evaluate release under physiologically relevant pH conditions and protein- and/or enzyme-containing media to better define the relationship between *in vitro* release and *in vivo* drug disposition. To further determine whether the release behavior was associated with matrix erosion, the hydrolytic degradation of different microsphere formulations was examined by monitoring weight loss over five weeks. Blank PLGA microspheres showed only limited mass loss, indicating the relatively slow degradation of the pristine polymer matrix under the tested conditions (Fig. 2i). By contrast, blank microspheres containing MgCO_3_ displayed markedly accelerated weight loss, and Leo-micro@MD showed a comparable degradation trend. Time-resolved SEM of Leo-micro@MD showed progressive surface roughening during weeks 1–2, followed by pore opening/local cavities during weeks 3–4 and a markedly porous, eroded surface by week 5 (Fig. S7). In parallel, GPC analysis revealed that the weight-average molecular weight of PLGA decreased by approximately 18% after five weeks (Fig. S8). These results suggest that MgCO_3_ played a dominant role in promoting matrix erosion in the optimized formulation, thereby contributing to the sustained release of Leo from Leo-micro@MD. Preliminary manufacturing reproducibility was assessed using three independently prepared batches of the selected Leo-micro@MD formulation. All three batches exhibited comparable spherical morphology by SEM and closely clustered drug-loading values around 19% (Fig. S9), supporting limited inter-batch variability at the current laboratory scale. Collectively, the high drug-loading capacity, prolonged release profile, and MgCO_3_-regulated degradation behavior demonstrate that the optimized Leo-micro@MD platform is suitable for long-acting Leo delivery.

### 3.2 Therapeutic effects of Leo-micro@MD on EMS *in vitro*

To establish the biological safety and therapeutic selectivity of the microcarriers, the *in vitro* cytocompatibility of Leo-micro@MD against normal endometrial stromal cells (nESCs) and ectopic endometrial stromal cells (eESCs) was systematically cross-evaluated. Firstly, the cell viability of primary nESC after 24 h incubation with Leo-micro@MD was evaluated by the Alamar Blue assay. As shown in Fig. S10a, Leo-micro@MD exhibited a cell survival rate of over 80% across a broad concentration range of 0–500 μg/mL, indicating its excellent cytocompatibility. Interestingly, Leo-micro@MD effectively inhibited eESC viability in a concentration-dependent manner, revealing a stark contrast where the long-acting microcarriers could preferentially eradicate pathological cells while avoiding toxic side effects on healthy counterparts (Fig. S10b).

Given that EMS shares several key pathological features with malignant tumors, including aggressiveness, local and distant progression, and resistance to apoptosis (Zhang et al., 2022), we subsequently investigated the inhibitory effect of Leo-micro@MD on the invasive capacity of eESCs using Transwell assays. As illustrated in Fig. 3a, the transmembrane invasion of eESCs were significantly elevated compared to nESCs. In contrast, eESC invasion was attenuated to different degrees after treatment with free Leo and Leo-micro@MD. Most notably, the number of invasive cells in the Leo-micro@MD group was attenuated to approximately one-third of that in the untreated model group (Fig. 3b). These findings underscore that Leo-micro@MD could effectively impede the invasion of eESCs, thereby theoretically hindering the metastasis and adhesion of ectopic endometrial tissue *in vivo*.

**Fig. 3:**
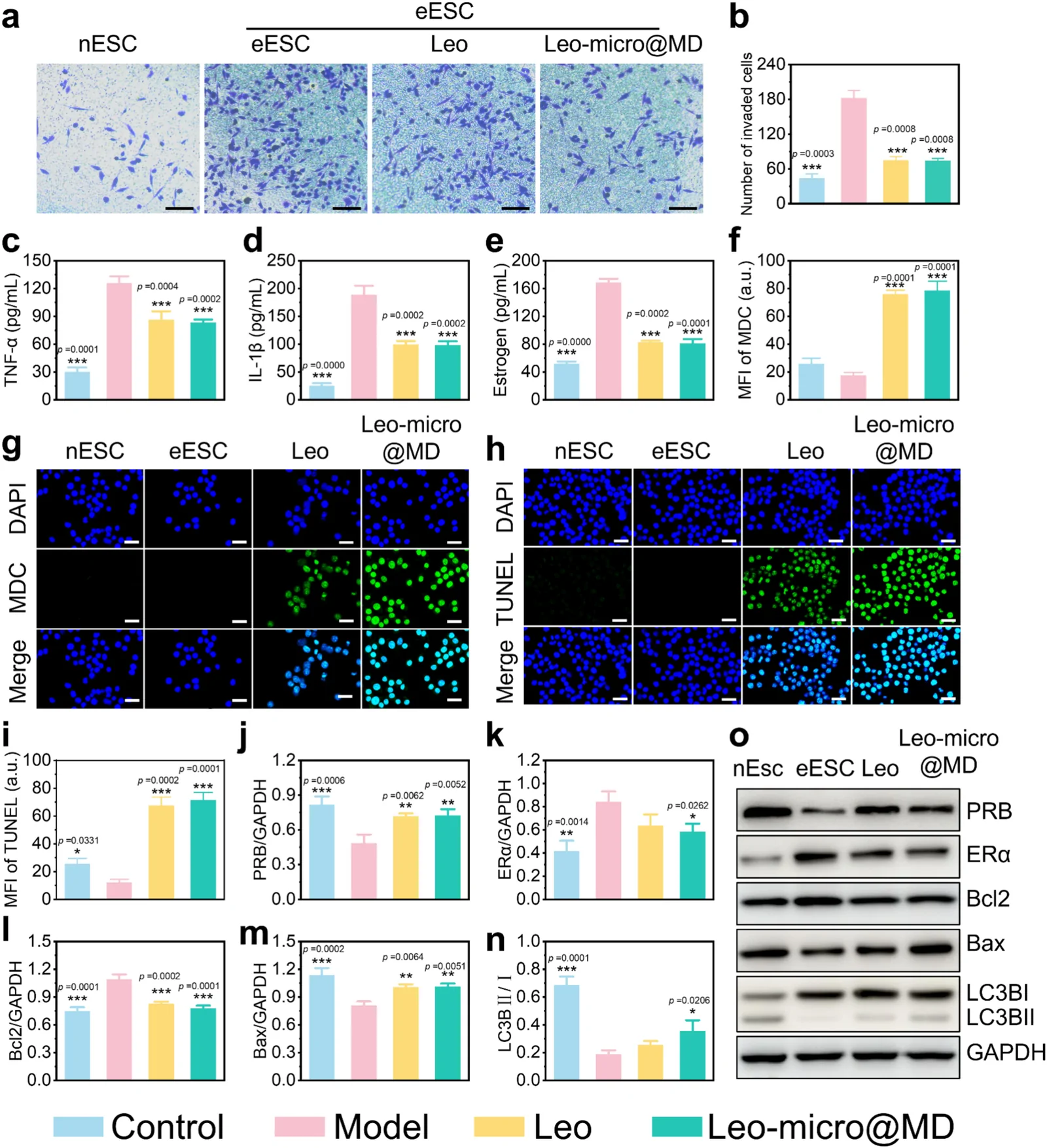
Therapeutic Effects of Leo-micro@MD on EMS *in vitro*. (a) Representative images of nESCs and eESCs invading across the transwell membrane in various groups and (b) the number of invaded cells per microscopic field. Scale bar = 100 μm. (c) TNF-α, (d) IL-1β, and (e) Estrogen secreted after different treatments. Autophagy enhancement effect by MDC staining. (f) Quantitative analysis of MFI in different groups, and (g) CLSM images, scale bar = 25 μm. Proapoptotic effect by TUNEL staining. (h) CLSM images, scale bar = 25 μm, and (i) quantitative analysis of MFI in different groups. Western blot of PRB, ERα, Bcl2, Bax, and LC3II/I produced by ESCs after different treatments. Quantitative analysis of (j) PRB, (k) ERα, (l) Bcl2, (m) Bax, and (n) LC3II/I expressions relative to GAPDH, and (o) the representative images. Data were expressed as mean ± SD, *n* = 3. * *p* < 0.05, **p < 0.01, ***p < 0.001, ****p < 0.0001 *vs* the Model group.

Furthermore, the systematic quantification of downstream inflammatory and endocrine markers validated that Leo-micro@MD could comprehensively reverse the pathological hyper-estrogenic and pro-inflammatory microenvironment. To be specific, a disordered pro-inflammatory microenvironment and aberrant endocrine signaling collaboratively drive the proliferation of ectopic lesions, with elevated levels of TNF-α and other pro-inflammatory factors closely associated with the pelvic pain and infertility commonly observed in patients with endometriosis (EMS). Compared with the untreated eESC model group, treatment with Leo-micro@MD significantly reduced the secretion levels of TNF-α and IL-1β to 66.2% and 52.1% of the model baseline, respectively (Fig. 3c, and d). A similar downward trajectory was observed in the IL-6 level (Fig. S11), further validating that Leo-micro@MD exhibits a strong anti-inflammatory activity. Since chronic inflammation frequently precipitates endocrine imbalance, local estrogen production was further quantified *via* ELISA. As shown in Fig. 3e, estrogen secretion in the eESC model was elevated to 3.2-fold of that in the control nESCs. However, this aberrant hyper-estrogenic state was successfully down-regulated to 49.0% after Leo-micro@MD treatment, signaling a profound restoration of homeostatic endocrine signaling.

To elucidate the molecular mechanisms underlying suppressed programmed cell death in endometriosis, the regulatory effects of Leo-micro@MD on the upstream hormone receptor axis were first evaluated. Pathologically, estrogen hyper-expression triggers an expression imbalance between estrogen receptor alpha (ERα) and progesterone receptor B (PRB), establishing an intracellular microenvironment that hinders the autophagy and apoptosis of ectopic endometrial stromal cells (eESCs). Western blot analysis revealed that untreated eESCs displayed decreased PRB expression and elevated ERα expression compared with normal endometrial stromal cells (nESCs), confirming a disrupted ERα/PRB axis (Fig. 3j-o). In contrast, free Leo partially restored this receptor balance by increasing PRB and reducing ERα. Notably, Leo-micro@MD showed a comparable PRB-restoring effect but produced a more evident reduction in ERα expression than free Leo, suggesting its superior efficacy in suppressing estrogen receptor signaling.

This hormone receptor remodeling subsequently reactivated the suppressed autophagic flux and triggered the intrinsic apoptotic cascade in eESCs. As delineated by the MDC assay, the nESC and eESC groups manifested only dim green fluorescence, indicative of a quiescent, low-autophagy state. In stark contrast, the green fluorescence of the Leo-micro@MD group was significantly enhanced, yielding a marked increase in Mean Fluorescence Intensity (MFI) (Fig. 3f, g). Paralleling this autophagic activation, TUNEL staining confirmed a homologous surge in programmed cell death (Fig. 3h, i). At the molecular level, Western blot analysis verified that Leo-micro@MD enhanced autophagy by significantly upregulating the LC3BII/I conversion ratio (Fig. 3n, o). This effect outperformed the moderate increase induced by free Leo, likely owing to the sustained release behavior and prolonged intracellular pharmacological action of the microdevice.

Concurrently, the expressions of downstream apoptosis-related proteins were modulated to counteract the abnormal survival advantage of eESCs. The untreated eESC model exhibited a survival-favored molecular state characterized by increased anti-apoptotic Bcl-2 and reduced pro-apoptotic Bax expression (Fig. 3l-o). Both free Leo and Leo-micro@MD reversed this pattern by downregulating Bcl-2 and upregulating Bax. Compared with free Leo, Leo-micro@MD caused a slightly stronger decrease in Bcl-2 and a comparable or modestly higher increase in Bax, consistent with the stronger TUNEL fluorescence observed. In summary, these findings substantiate that the Leo-micro@MD platform effectively delays endometriosis progression. By repairing the TNF-α-induced estrogen and progesterone receptor imbalances, Leo-micro@MD reverses low autophagy and ultimately drives a highly efficient apoptotic cascade to suppress eESC survival.

### 3.3 Therapeutic effects and biosafety evaluation of Leo-micro@MD in EMS mice

Encouraged by the multifaceted therapeutic efficacy and favorable cytocompatibility verified *in vitro*, we next translated our investigation *in vivo* to systematically evaluate the therapeutic potential of Leo-micro@MD in EMS. After establishing the EMS model by injecting endometrial fragments into recipient mice, we conducted a 28-day treatment and subsequently dissected the mice to assess the weight and volume of the ectopic lesions. The untreated model mice developed multiple ectopic lesions with relatively large lesion sizes and an obvious vascularized tissue appearance, confirming successful EMS model establishment (Fig. 4a). Free Leo reduced the overall lesion burden to a certain extent, whereas Leo-micro@MD produced the most evident lesion regression, with visibly smaller and fewer ectopic nodules. This macroscopic difference suggested that microsphere-mediated delivery strengthened the *in vivo* therapeutic performance of Leo. Quantitative analysis further confirmed the strong lesion-suppressive effect of Leo-micro@MD. The untreated model group showed approximately 6.3 ectopic lesions per mouse, whereas free Leo reduced the lesion number to approximately 5.1. Leo-micro@MD further decreased the lesion number to approximately 4.1, corresponding to an approximate 35% reduction compared with the model group and an additional 20% reduction compared with free Leo (Fig. 4b). More importantly, Leo-micro@MD exerted a stronger inhibitory effect on lesion growth than on lesion number. The ectopic lesion volume from the Leo-micro@MD group underwent an approximately 70% sharp decrease compared to that in the model group (Fig. 4c). A similar trend was observed for lesion weight. The average lesion weight decreased from approximately 0.11 g in the model group to approximately 0.085 g after free Leo treatment, whereas Leo-micro@MD further reduced the lesion weight to approximately 0.055 g, achieving nearly a 50% reduction compared with the model group and an additional 35% decrease compared with free Leo (Fig. 4d). These data demonstrate that Leo-micro@MD more effectively suppresses ectopic lesion progression than free Leo, particularly in terms of lesion volume and weight.

**Fig. 4:**
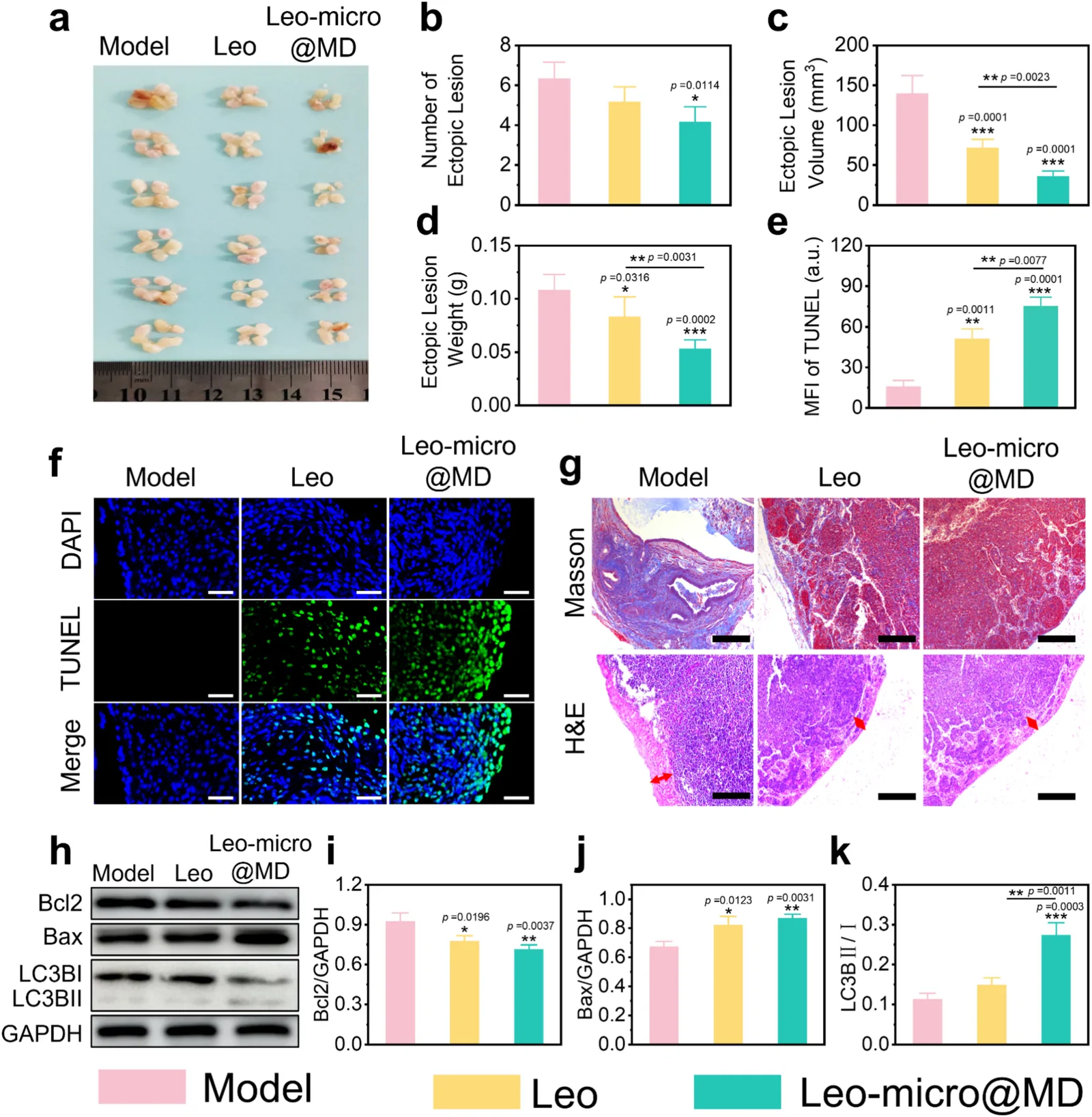
Therapeutic effects of Leo-micro@MD in EMS mice. (a) Representative images of ectopic lesions in mice with endometriosis after different treatments. (b) The number, (c) volume, and (d) weight of ectopic lesions after different treatments. (e) Quantitative analysis and (f) representative images of fluorescence intensity of TUNEL staining of ectopic lesions. (scale bar = 50 μm) (g) Masson staining and H&E staining images of ectopic lesion sections from different treatment groups (scale bar = 50 μm). The red arrows indicate the thickness of the ectopic cyst wall. (h) Western blot analysis of Bcl2, Bax, and LC3II/I levels in ectopic lesions. The relative protein levels of (i) Bax, (j) Bcl2, and (k) LC3II/I. Data were expressed as mean ± SD. *n* = 6. * *p* < 0.05, ∗∗ *p* < 0.01, ∗∗∗ *p* < 0.0010 *vs* the Model group. (For interpretation of the references to colour in this figure legend, the reader is referred to the web version of this article.)

Since resistance to apoptosis is a key pathological feature that enables ectopic endometrial cells to survive outside the uterine cavity, TUNEL staining was performed to determine whether lesion regression was associated with enhanced programmed cell death (Zhong et al., 2025). The untreated model lesions displayed only weak TUNEL-positive fluorescence, indicating limited spontaneous apoptosis within ectopic tissues (Fig. 4e, f). Free Leo markedly increased TUNEL signals, suggesting that Leo promoted apoptotic elimination of lesion cells. Notably, Leo-micro@MD induced the strongest TUNEL fluorescence among all groups. Quantitative analysis showed that Leo-micro@MD enhanced the apoptotic signal by approximately 4.4-fold compared with the model group and by approximately 1.5-fold compared with free Leo. These findings indicate that the superior lesion regression achieved by Leo-micro@MD is closely associated with more efficient activation of apoptosis in ectopic tissues.

To further demonstrate the structural remodeling and anti-fibrotic capacity of the microcarriers at the tissue level, histological evaluations were conducted using H&E and Masson trichrome staining on the harvested lesion slices (Fig. 4g). EMS lesions are frequently accompanied by gland-like structures, stromal expansion, inflammatory infiltration, and collagen-rich fibrosis, all of which contribute to lesion persistence, adhesions, pelvic pain, and impaired tissue function (Karp and Stratton, 2023). Masson trichrome staining revealed abundant collagen deposition in the untreated model lesions, indicating marked fibrotic remodeling. In contrast, collagen accumulation was reduced after free Leo treatment and was further diminished in the Leo-micro@MD group. In parallel, H&E staining further delineated the internal structural architecture of each group, showing that the outer boundary of the ectopic lesions in the untreated Model group was heavily enveloped by dense fibrotic tissue. Quantitative tracking of the surrounding fibrotic borders demonstrated that intervention with free Leo and Leo-micro@MD significantly attenuated the wall thickness of the EMS lesions, thereby confirming their potent anti-fibrotic properties *in vivo*.

To further verify the molecular mechanism underlying Leo-micro@MD-induced lesion regression, apoptosis-related and autophagy-related proteins were analyzed thereafter by Western blotting. Therefore, a decrease in Bcl-2 together with an increase in Bax indicates a shift from survival toward apoptotic clearance. As shown in Fig. 4h–j, free Leo moderately reduced Bcl-2 expression and increased Bax expression compared with the model group. Leo-micro@MD produced a stronger pro-apoptotic protein profile, resulting in a 70% reduction in Bcl-2 expression and a 1.5-fold increase in Bax expression compared to the Model group. Compared with free Leo, Leo-micro@MD further reduced Bcl-2 and slightly enhanced Bax expression, consistent with the stronger TUNEL-positive apoptotic response observed in lesion tissues. Autophagy activation was further examined by assessing the LC3B-II/I ratio. Dysregulated autophagy contributes to the abnormal survival and persistence of ectopic endometrial cells in EMS, whereas reactivation of autophagy can cooperate with apoptosis to promote lesion regression. Western blot analysis showed that free Leo slightly increased the LC3B-II/I ratio compared with the model group, whereas Leo-micro@MD induced a much stronger elevation (Fig. 4h, k). The quantitative results showed that after treatment with Leo-micro@MD, the ratio of LC3B-II/I increased further to 2.5 times that of the Model group. Collectively, these findings substantiate that Leo-micro@MD efficiently eradicates ectopic lesions *via* the synchronized reactivation of autophagy and apoptosis *in vivo*.

To further clarify how Leo-micro@MD remodeled the pathological microenvironment of EMS *in vivo*, inflammatory cytokine production and endocrine receptor homeostasis were systematically evaluated in ectopic lesions. Since chronic inflammation is a central driver of EMS progression, TNF-α expression in ectopic lesions was first examined by immunohistochemistry. TNF-α is a key pro-inflammatory cytokine that promotes ectopic stromal cell survival, adhesion, proliferation, angiogenesis, and pain-associated inflammatory signaling in EMS (Dai et al., 2025). As shown in Fig. 5a, TNF-α staining was barely detectable in the control tissues but was markedly increased in the untreated model lesions, confirming the establishment of an inflammatory lesion microenvironment. Free Leo significantly reduced the TNF-α-positive area to approximately 40% of the model level (Fig. 5b). Notably, Leo-micro@MD produced a stronger inhibitory effect, further decreasing tissue TNF-α expression to approximately 28.7% of the model level, indicating that microsphere-mediated delivery enhanced the anti-inflammatory activity of Leo within ectopic lesions.

**Fig. 5:**
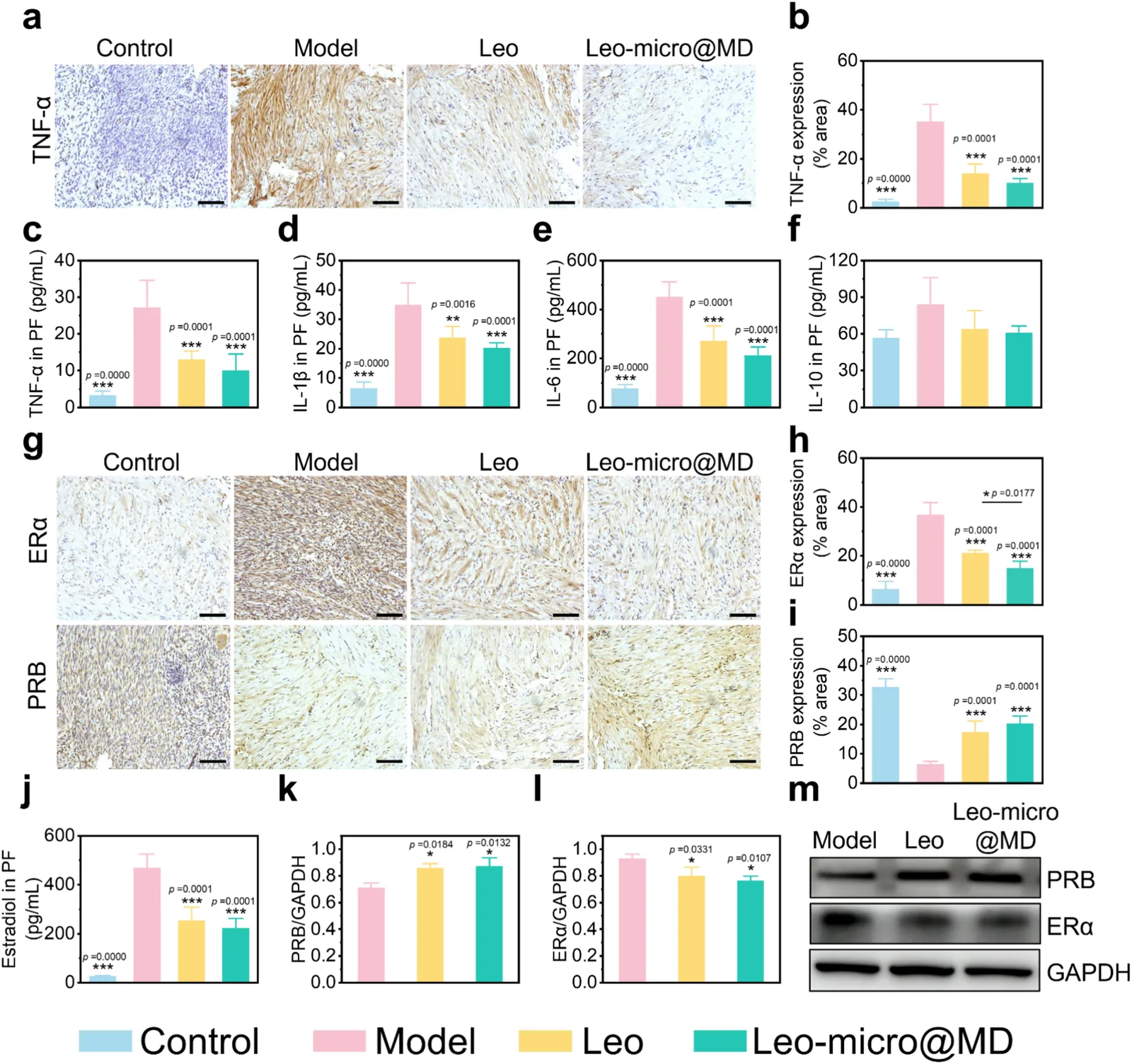
The effect of remodeling the inflammatory and endocrine microenvironment of ectopic lesions *in vivo*. Leo-micro@MD (a) Immunohistochemical staining images of TNF-α. (scale bar = 100 μm). (b) Quantitative analysis of TNF-α expression in EMS lesions. The levels of (c) TNF-α, (d) IL-1β, (e) IL-6, and (f) IL-10 production in mouse peritoneal fluid after different treatments. (g) Immunohistochemical staining images of ERα and PRB. (scale bar = 100 μm). Quantitative analysis of (h) ERα, and (i) PRB expression in EMS lesions. (j) The levels of estradiol production in mice peritoneal fluid after different treatments. The relative protein levels of (k) PRB and (l) Erα, and (m) Western blot analysis of Erα and PRB levels in ectopic lesions. Data were expressed as mean ± SD. *n* = 6. ∗*p* < 0.05, ∗∗*p* < 0.01, ∗∗∗*p* < 0.001 *vs* the Model group.

The inflammatory status of the peritoneal microenvironment was further quantified by measuring cytokine levels in peritoneal fluid (PF). The peritoneal cavity is the primary site for ectopic lesion implantation and expansion; therefore, inflammatory mediators in PF directly reflect the pathological milieu that supports lesion persistence. Consistent with the IHC results, TNF-α concentration in PF was markedly elevated in the model group but was reduced after both treatments (Fig. 5c). Free Leo decreased PF TNF-α to approximately 48.1% of the model level, whereas Leo-micro@MD further reduced it to approximately 36.9%. In parallel, IL-1β is another inflammatory cytokine involved in pain sensitization, immune cell recruitment, and lesion progression, and it was also substantially increased in the model group (Fig. 5d) (Titiz et al., 2024). Free Leo reduced IL-1β to approximately 65–70% of the model level, while Leo-micro@MD further decreased it to nearly 55%, suggesting a stronger suppression of IL-1β-driven inflammatory amplification. Similarly, IL-6, which contributes to ectopic cell proliferation, angiogenesis, and persistent inflammatory signaling in EMS, was sharply increased in PF after EMS induction (Fig. 5e). From quantitative assessment, the free Leo treatment reduced IL-6 to approximately 60% of the model level, whereas Leo-micro@MD further decreased it to approximately 45%, indicating a more effective attenuation of IL-6-associated inflammatory activation (Zhang et al., 2026). In comparison, IL-10 showed a moderate downward trend after treatment (Fig. 5f), as elevated IL-10 in EMS may also reflect immune dysregulation and has been associated with lesion persistence and fibrotic remodeling. Therefore, the partial normalization of IL-10 after Leo-micro@MD treatment suggests a broader rebalancing of the peritoneal immune microenvironment.

To elucidate the systemic endocrine modulation *in vivo*, the regulatory effects of Leo-micro@MD on the hyper-estrogenic signaling and progesterone resistance within the ectopic microenvironment were systematically evaluated. Since the endocrine microenvironment imbalance of ectopic endometrium is fundamentally characterized by elevated estrogen signaling and progesterone resistance that promote lesion growth (Blair, 2024), we evaluated the capacity of Leo-micro@MD to restore hormone receptor homeostasis *in vivo*. As shown in Fig. 5g, the immunohistochemical results of ectopic lesions showed that treatment with both free Leo and Leo-micro@MD significantly suppressed the expression of the estrogen receptor ERα while up-regulating the progesterone receptor PRB. Among them, the therapeutic effect of the Leo-micro@MD group was most evident, restoring PRB and ERα levels to 313.7% and 40.7% of the Model group, respectively (Fig. 5h, i). At the same time, ELISA results verified that EMS onset induced a sharp increase in estrogen in the peritoneal fluid, whereas Leo-micro@MD treatment successfully down-regulated this hyper-estrogenic state to only 30.4% of the Model group value (Fig. 5j). This receptor remodeling was further confirmed by Western blot analysis, which demonstrated a consistent down-regulation of ERα and up-regulation of PRB in the treated ectopic tissues (Fig. 5k-m), thereby proving that Leo-micro@MD intervention could successfully restore the homeostatic Erα/PRB balance *in vivo*.

In summary, all these results indicated that Leo-micro@MD treatment reversed the imbalance of ERa and PRB in EMS ectopic lesions by inhibiting the production of TNF-α and other inflammatory factors, thus subsequently promoting the induction of autophagy in ectopic lesions, leading to apoptosis of ectopic endometrial tissue, and therefore effectively alleviating EMS progression.

The preliminary *in vivo* biosafety of Leo-micro@MD was further evaluated to determine whether its therapeutic efficacy in EMS mice was accompanied by detectable systemic toxicity. The tolerance of Leo-micro@MD was evaluated through serum biochemical analysis, weight monitoring, organ index assessment, and histological examination of the major organs. The liver and kidney function of the Leo-micro@MD group were analyzed by serological biochemistry. The results showed no significant difference in the levels of alanine aminotransferase (ALT), aspartate transaminase (AST), creatinine (CRE), and blood urea nitrogen (BUN) between the control group and the Leo-micro@MD group, suggesting that the liver and kidney function were well preserved after treatment (Fig. S12). Consistently, no apparent body-weight loss was observed during the treatment period, indicating that Leo-micro@MD was well tolerated *in vivo* (Fig. S13). Spleen index and liver index of EMS mice were measured at the end of treatment. Compared with the healthy mice, there was no significant difference between the free Leo and the Leo-micro@MD groups, further suggesting that Leo-micro@MD did not cause obvious immune-organ enlargement or liver-associated toxicity (Fig. S14). Histological examination of the heart, liver, spleen, lung, and kidney by H&E staining revealed no evident inflammatory infiltration, tissue necrosis, or structural damage in the Leo-micro@MD group (Fig. S15). Collectively, these results demonstrate that Leo-micro@MD exhibited favorable preliminary biosafety in EMS mice, supporting its further evaluation as a long-acting therapeutic platform for EMS treatment.

### 3.4 Therapeutic Effects of Leo-micro@MD on RA *in vitro*

Given that endometriosis and rheumatoid arthritis share strikingly homologous pathological hallmarks since both conditions are predominantly driven by a chronic, hyper-activated pro-inflammatory microenvironment and aggressive cellular invasion, we systematically extended the therapeutic evaluation of the Leo-micro@MD microparticles to RA-associated cellular models. First, human rheumatoid fibroblast-like synoviocytes, especially the MH7A cells, were employed to evaluate the intrinsic cytotoxicity of the developed Leo-micro@MD platform. After a 24 h incubation across a broad equivalent Leo dose range spanning from 1 to 1000 μM, the viability of the MH7A cells remained consistently above 90%, which demonstrated a highly favorable cytocompatibility profile (Fig. S16).

Subsequently, the *in vitro* anti-inflammatory and anti-invasive activities of Leo-micro@MD were systematically evaluated in LPS-stimulated MH7A cells to mimic key pathological events in RA. Oxidative stress is an upstream amplifier of RA inflammation, as excessive reactive oxygen species (ROS) can activate NF-κB and inflammasome-related signaling, promote cytokine secretion, aggravate synovial hyperplasia, and contribute to cartilage and bone destruction (Kang et al., 2026). Therefore, intracellular ROS generation was first examined using DCFH-DA staining. As shown in Fig. 6a, LPS stimulation markedly increased green fluorescence intensity compared with the Control group, indicating robust ROS accumulation. Free Leo substantially reduced ROS generation, decreasing the ROS mean fluorescence intensity to approximately 50% of the Model group level. Notably, Leo-micro@MD further lowered ROS accumulation to nearly 45% of the model level, showing a slightly stronger antioxidant effect than free Leo (Fig. 6b). These results suggested that Leo-micro@MD effectively attenuates LPS-induced oxidative stress, which might help interrupt the ROS-driven inflammatory amplification loop in RA.

**Fig. 6:**
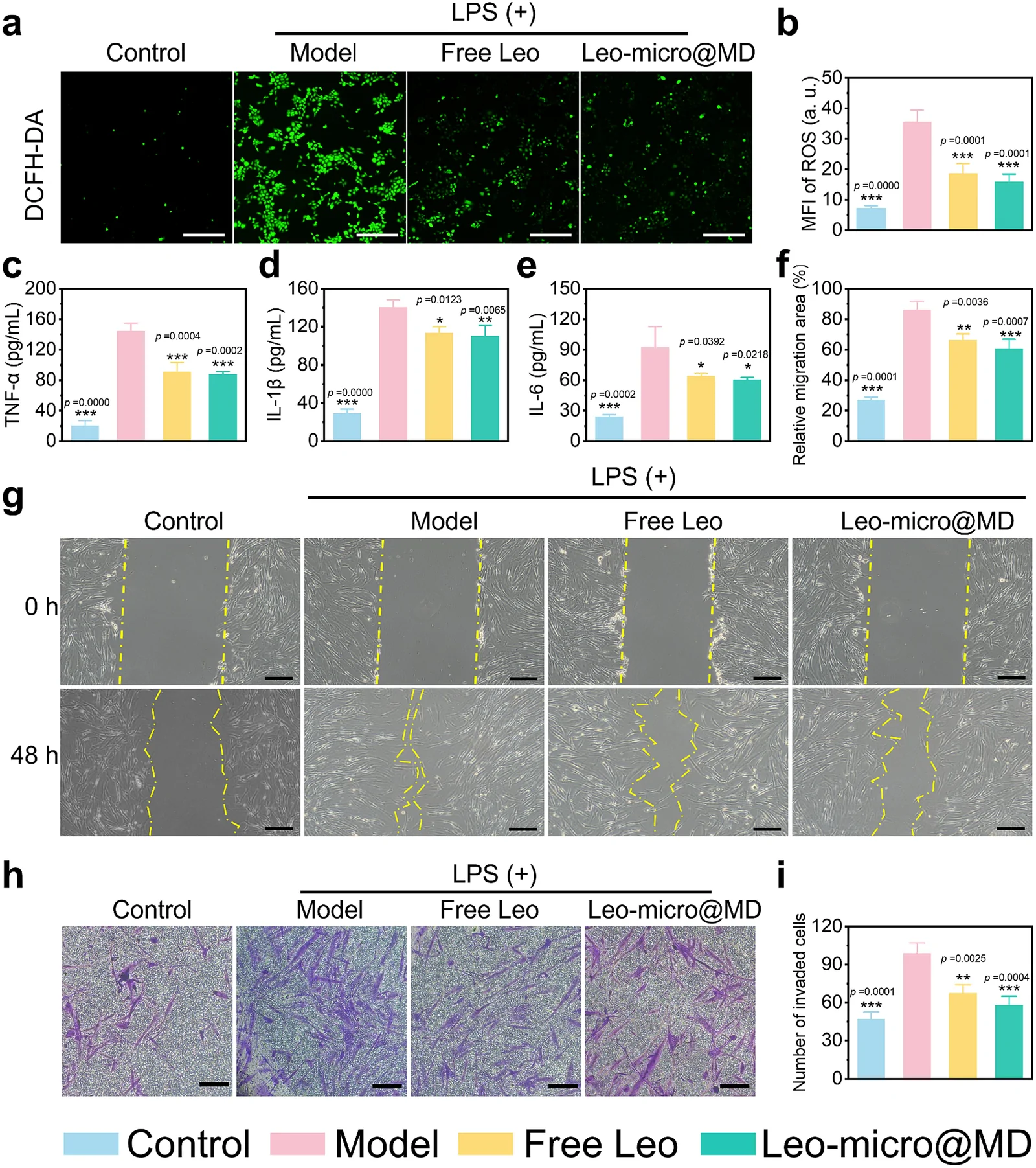
Therapeutic Effects of Leo-micro@MD on RA *in vitro*. (a) Fluorescence imaging and (b) the relative level of intracellular ROS in MH7A cells with different treatments. Quantification of inflammatory cytokines (c) TNF-α, (d) IL-1β, and (e) IL-6 secreted by RAW264.7 cells. (f) The relative migration area and (g) typical images of MH7A cells at 0 h and 48 h. Scale bar = 200 μm. (h) Representative images of MH7A cells invading across the transwell membrane in various groups and (i) the number of invaded cells per microscopic field. Scale bar = 100 μm. Data were expressed as mean ± SD, *n* = 3. * *p* < 0.05, ** *p* < 0.01, *** *p* < 0.001, **** *p* < 0.0001 *vs* the Model group.

To systematically investigate the *in vitro* anti-inflammatory potency of the microcarriers within a biomimetic arthritic environment, the regulatory effects of Leo-micro@MD on macrophage-derived cytokine cascades were comprehensively quantified *via* ELISA. Since pro-inflammatory cytokines serve as pivotal deleterious drivers in the pathogenesis of rheumatoid arthritis, leading to continuous articular inflammation that inevitably precipitates irreversible joint damage (Brown et al., 2024), LPS-activated RAW 264.7 macrophages were utilized as a pathological model. Compared to the control group, LPS stimulation dramatically enhanced the secretion of TNF-α, IL-1β, and IL-6, confirming the successful establishment of a pro-inflammatory phenotype (Fig. 6c–e). Free Leo significantly reduced these cytokines, demonstrating its intrinsic anti-inflammatory activity. Leo-micro@MD produced a comparable or slightly stronger inhibitory effect, reducing TNF-α, IL-1β, and IL-6 to approximately 60.6%, 77.8%, and 65.5% of the model levels, respectively. In particular, TNF-α and IL-6 levels in the Leo-micro@MD group were slightly lower than those in the free Leo group, indicating that microsphere-mediated delivery preserved and modestly enhanced the anti-inflammatory efficacy of Leo. Concurrently, the secretion of IL-10, which acts as a vital immunomodulatory cytokine with established chondroprotective and anti-erosive properties within RA joints, was substantially up-regulated in both intervention groups (Fig. S17) (Zhang et al., 2025). This synchronized suppression of destructive cascades and the concurrent elevation of protective regulatory factors collectively verified the potent, broad-spectrum anti-inflammatory repertoire of the Leo-micro@MD platform.

Since the tumor-like migration and invasion of activated fibroblast-like synoviocytes directly orchestrate pannus formation and subsequent cartilage erosion, the capacity of Leo-micro@MD to impede MH7A motility was thoroughly charted *via* wound healing and Transwell assays. As shown in Fig. 6f-g, the LPS-induced model group exhibited rapid wound closure after 48 h, with the relative migration area increasing to approximately 90%, which was more than threefold higher than that of the control group. Leo significantly restrained MH7A migration, reducing the relative migration area to approximately 75% of the model level. Leo-micro@MD further decreased the relative migration area to approximately 70% of the model level, corresponding to an approximately 30% reduction in migratory capacity compared with the model group. The stronger suppression observed in the Leo-micro@MD group indicated that the microsphere formulation more effectively limited the migration of activated FLSs. The anti-invasive effect of Leo-micro@MD was further verified by a Transwell invasion assay. FLS invasion is a critical cellular behavior in RA because invasive FLSs penetrate cartilage and subchondral bone, secrete matrix metalloproteinases, and directly contribute to irreversible structural damage (Kurowska-Stolarska and Alivernini, 2022). As shown in Fig. 6h and i, LPS stimulation markedly enhanced the number of invaded MH7A cells, reaching nearly twice that of the Control group. Free Leo significantly reduced the number of invaded cells to approximately 70% of the model level. Leo-micro@MD produced a more pronounced inhibitory effect, decreasing the invaded cell number to nearly 60% of the model level. Compared with free Leo, Leo-micro@MD further lowered the invaded cell number by approximately 15%, suggesting that the sustained-release microcomposite depot system more efficiently attenuated the aggressive phenotype of activated FLSs. In summary, these holistic *in vitro* results substantiate that Leo-micro@MD possessed remarkable cytocompatibility while simultaneously exerting dual-functional profiles by alleviating inflammatory surges and halting FLS migration and invasion, thereby highlighting its profound translational potential to effectively mitigate RA progression *in vivo*.

### 3.5 Therapeutic effects and biosafety evaluation of Leo-micro@MD in RA with AIA rats

Pharmacokinetic analysis demonstrated markedly prolonged systemic exposure following Leo-micro@MD administration (Fig. S18 and Table S2). Compared with free Leo, microsphere delivery extended the apparent terminal half-life (T_1/2_) and the mean residence time (MRT_0-∞_) by approximately 12.4- and 34.7-fold, respectively, while maintaining measurable circulating Leo over one week. The plasma profile was multiphasic, comprising an early exposure phase followed by a delayed secondary elevation. Similar biphasic or multiphasic systemic profiles have been reported for long-acting microsphere and subcutaneous depot formulations, reflecting the contribution of temporally distinct drug-input processes (Park et al., 2022; Wan et al., 2023). Together with the *in vitro* release results, these pharmacokinetic findings support substantially prolonged formulation-mediated systemic exposure of Leo. Numerical deconvolution further resolved this biphasic pharmacokinetic behavior into a relatively limited early input followed by a broader delayed formulation-mediated input phase (Fig. S19). Correspondingly, the cumulative fraction input increased progressively throughout the observation period, further supporting sustained and temporally heterogeneous systemic drug input from the microsphere depot (Wan et al., 2022; Wang et al., 2025). Tissue-level exposure was not assessed in this study, and tissue-specific accumulation was therefore not inferred from plasma data. Future work will use validated LC–MS/MS methods to establish time-resolved tissue pharmacokinetics of Leo-micro@MD in ectopic endometriotic lesions, inflamed joint/synovial tissues, and major organs, thereby linking systemic exposure to local accumulation at target disease sites.

Building upon the profound therapeutic outcomes achieved in the EMS model, we further expanded our *in vivo* investigations to explore the potential of Leo-micro@MD in treating RA. To systematically evaluate this anti-arthritic efficacy, an adjuvant-induced arthritis (AIA) rat model was successfully established. Following disease onset, the untreated Model group exhibited severe hind paw swelling and erythema, which were effectively alleviated across the treatment cohorts (Fig. 7a). More precisely, the clinical inflammation scores in the Leo-micro@MD group were significantly reduced to 55.5% and 49.1% of those in the free Leo and Model groups, respectively (Fig. 7b). Consistent with these observations, the progressive increase in hind paw volume was prominently suppressed after free Leo and Leo-micro@MD treatments by day 30 (Fig. 7c). This markedly enhanced anti-rheumatic activity of the Leo-micro@MD formulation can be primarily attributed to its stable plasma concentration and prolonged retention. Furthermore, as continuous RA progression inherently impedes normal dietary intake and induces cachexia, rats in the Model group experienced a sharp decline in body weight (Fig. 7d). However, treatment with free Leo and Leo-micro@MD successfully reversed this progressive weight loss.

**Fig. 7:**
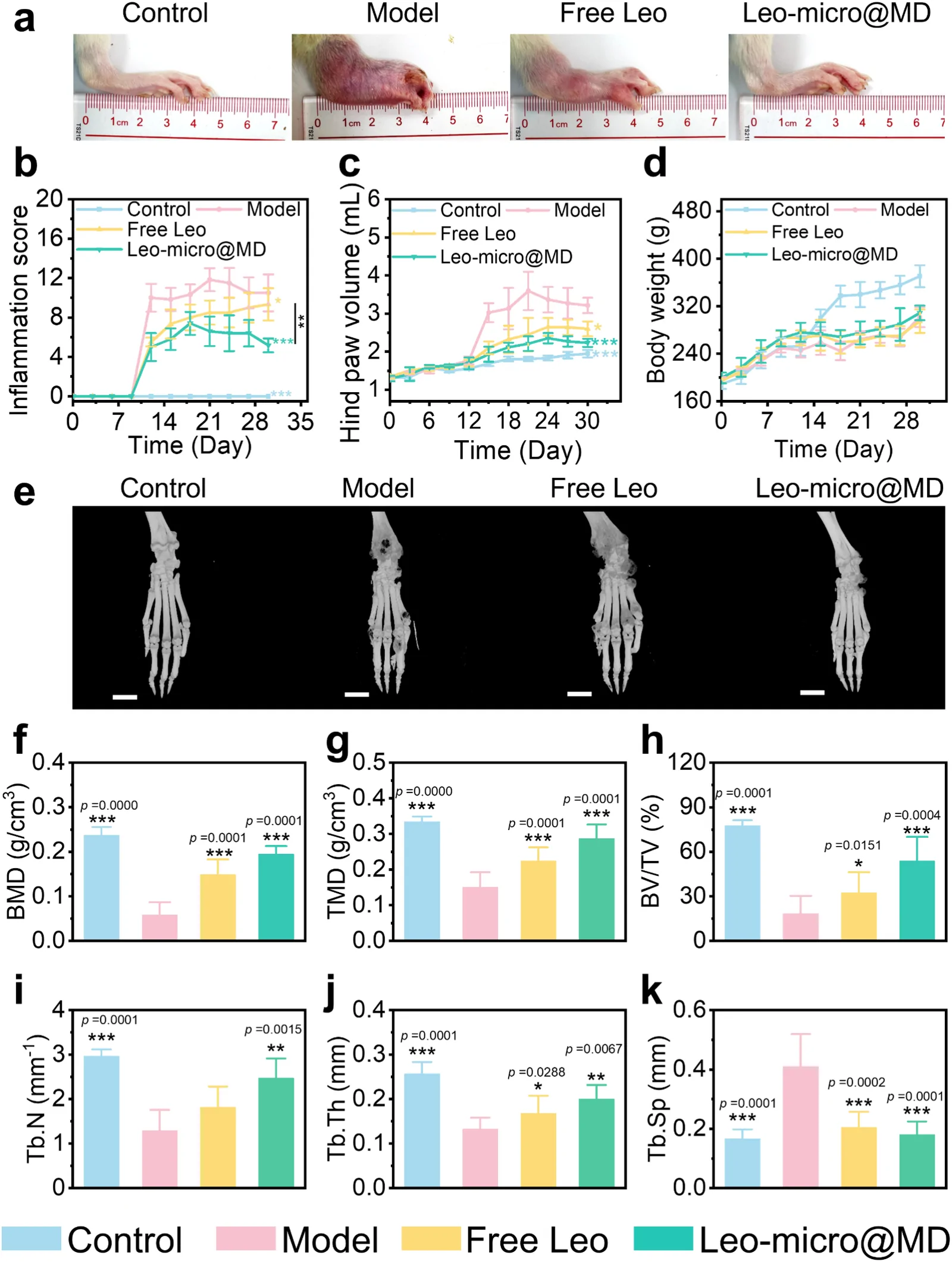
Therapeutic Effects of Leo-micro@MD in RA with AIA rats. (a) Representative images of hind paws. (b) Inflammation score, (c) hind paw volume, and (d) body weight of rats. (e) The hind paws scanned by micro-CT. Scale bar = 2.5 mm. Histomorphometry parameters were obtained from analyses of the micro-CT data. (f) BMD: bone mineral density, (g) TMD: tissue mineral density, (h) BV/TV: bone volume fraction (i) Tb.N: Trabecular number, (j) Tb.Th: Trabecular thickness, and (k) Tb.Sp: Trabecular bone spacing of the rats. Data were expressed as mean ± SD. *n* = 6. ∗*p* < 0.05, ∗∗*p* < 0.01, ∗∗∗*p* < 0.001.

Since bone erosion represents a pivotal pathological hallmark of progressive RA, micro-CT imaging was employed to comprehensively assess the structural integrity of the affected joints (Hong et al., 2026). As depicted in Fig. 7e, severe bone destruction and cortical surface erosion were prevalent in the untreated Model group, whereas the Leo-micro@MD cohort exhibited only minimal erosive damage. Further anatomical evaluation revealed substantial trabecular degradation within the tibia, fibula, and astragalus of the AIA rats, which was remarkably prevented by Leo-micro@MD treatment (Fig. S20). In addition, micro-CT data were employed to quantify trabecular injury across different groups by tracking reductions in BMD and TMD, which predict subsequent joint injury and serve as surrogate indicators of clinical RA progression (Tang et al., 2024a). As expected, both BMD and TMD in the hindlimbs of AIA rats were significantly reduced compared to the control group, suggesting severe osteoporosis (Fig. 7f, g). In stark contrast, BMD in the Leo-micro@MD group increased to approximately three times that of the Model group. Similarly, TMD also rose to a level close to that of the healthy control group. Moreover, the Leo-micro@MD formulation significantly improved bone degradation by up-regulating the bone volume fraction while decreasing total porosity (Fig. 7h, S21a). Compared with the control group, AIA rats displayed a dramatic decrease in bone volume fraction (BV/TV) and a marked increase in total porosity, indicating extensive trabecular bone destruction. The BV/TV ratio of the Leo-micro@MD group was approximately twice as high as that of the Free Leo group. Additionally, after treatment with Leo-micro@MD, the total porosity decreased to half of that of the model group, indicating a clear restoration of bone compactness and structural continuity. Trabecular microarchitecture was then evaluated using trabecular number (Tb.N), trabecular thickness (Tb.Th), trabecular separation (Tb.Sp), and structural model index (SMI). AIA induction markedly reduced Tb.N and Tb.Th while increasing Tb.Sp and SMI, suggesting that the trabecular network became thinner, more widely separated, and shifted from a plate-like structure toward a rod-like, mechanically weaker architecture (Fig. 7i-k, S21b). Leo-micro@MD substantially reversed these pathological changes. The improvements were consistently more pronounced than those observed in the free Leo group, demonstrating that Leo-micro@MD more effectively preserved trabecular integrity and attenuated RA-associated osteolytic deterioration.

To decode the systemic immunomodulatory and matrix-stabilizing efficacy of the formulation, key serum cytokines and matrix metalloproteinases were quantified *via* ELISA. In RA progression, TNF-α and IL-1β are overproduced in the synovium and are the major pro-inflammatory cytokines. In parallel, IL-6 plays a key role in inflammatory processes, osteoclast-mediated bone resorption, and synovitis. On the contrary, IL-10 acts as a crucial anti-inflammatory regulator (Zhang et al., 2023). Concretely, serum ELISA analysis revealed that the abnormally elevated concentrations of TNF-α, IL-1β, and IL-6 in the Model group were significantly suppressed across all treatment groups, with the Leo-micro@MD group achieving the most profound reduction in these pro-inflammatory mediators (Fig. 8a-c). Concurrently, the expression of the protective cytokine IL-10 was significantly up-regulated following Leo-micro@MD treatment, thereby establishing a favorable microenvironment to inhibit the inflammatory cascade and promote tissue remodeling (Fig. 8d). Furthermore, the critical homeostatic balance between MMP-3, a key marker of cartilage injury, and its endogenous inhibitor TIMP-1 was evaluated (Liu et al., 2022). Compared to the elevated MMP-3 and depleted TIMP-1 levels observed in the untreated AIA rats, Leo-micro@MD intervention successfully normalized both parameters to levels comparable to the healthy control (Fig. 8e, f), confirming its capacity to halt matrix degradation.

**Fig. 8:**
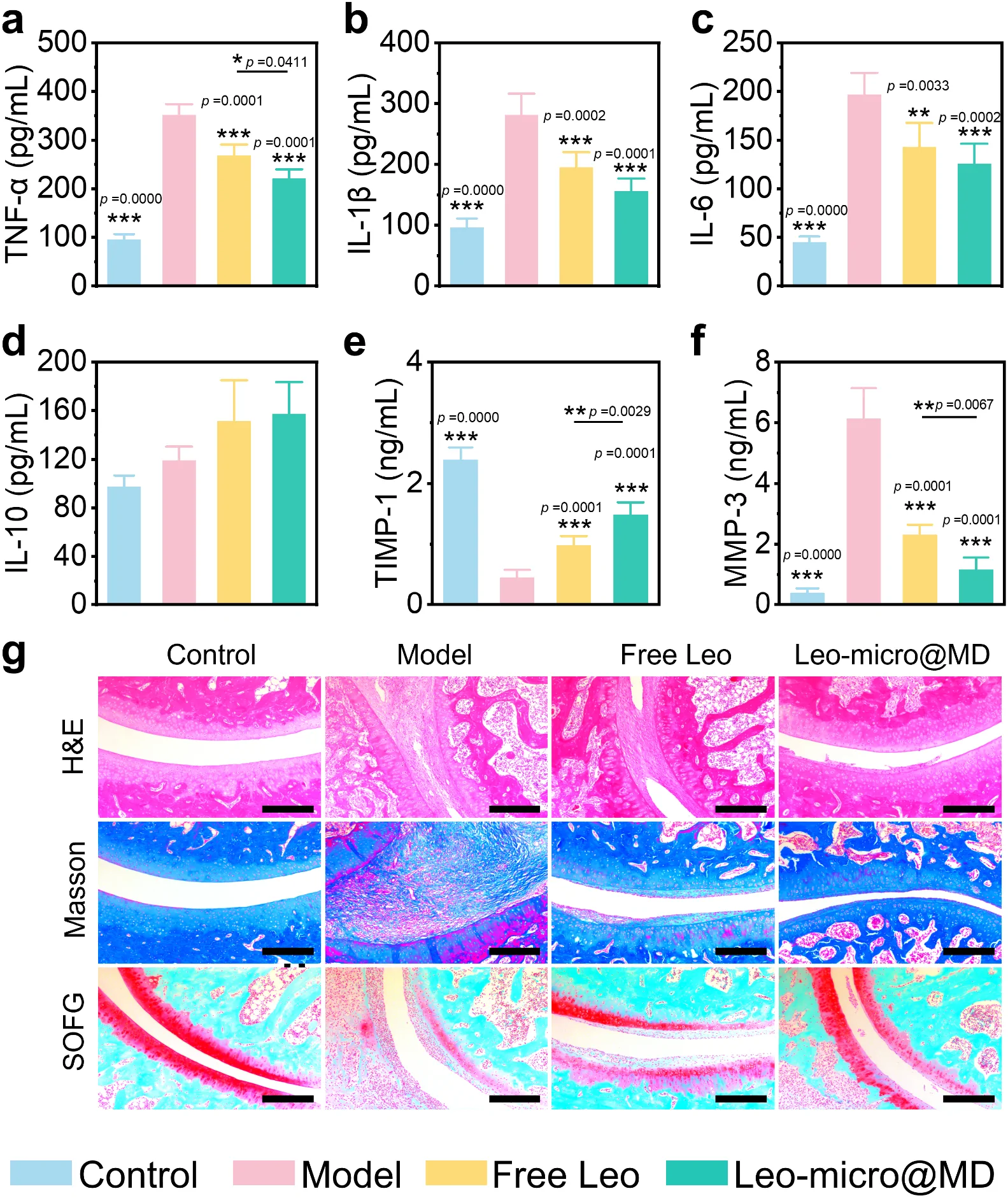
Therapeutic Effects of Leo-micro@MD in RA with AIA rats. Expression levels of inflammatory cytokines (a) TNF-α, (b) IL-1β, (c) IL-6, (d) IL-10, (e) MMP-3, and (f) TIMP-1 in serum measured by ELISA. (g) Histological analysis of joint tissues: H&E, Masson, and Safranin O-fast green staining (SOFG). Scale bar = 300 μm. Data were expressed as mean ± SD. *n* = 6. ∗*p* < 0.05, ∗∗*p* < 0.01, ∗∗∗*p* < 0.001. (For interpretation of the references to colour in this figure legend, the reader is referred to the web version of this article.)

To visually corroborate these protective effects at the microscopic tissue level, comprehensive histological assessments of the ankle joints were performed using H&E, Masson's trichrome, and SOFG staining (Fig. 8g). In detail, H&*E*-stained sections of the untreated Model group exhibited severe synovial hyperplasia, marked chondrocyte depletion, joint cavity stenosis, and massive inflammatory cell infiltration. In sharp contrast, the Leo-micro@MD group significantly reduced cartilage erosion, synovial hyperplasia, and inflammatory infiltration. In addition, the joint cavity of the Leo-micro@MD group was smooth with minimal neutrophil infiltration, confirming the optimal therapeutic effect. Since collagen constitutes the primary structural scaffold of the articular cartilage matrix, Masson's trichrome staining was evaluated, revealing extensive collagen loss in the Model group that was prominently restored by intense collagen deposition after the Leo-micro@MD treatment. Consistently, SOFG staining further highlighted these chondroprotective properties. The severe proteoglycan loss and nearly completely worn cartilage (blue-green) in the Model group were strikingly reversed in the Leo-micro@MD group, which maintained clear anatomical boundaries and a thick layer of intact cartilage (red). Taken together, these histological findings confirm that Leo-micro@MD effectively halts joint degeneration and facilitates cartilage tissue repair *in vivo*.

The systemic tolerability of Leo-micro@MD was further examined in RA rats to exclude potential treatment-related toxicity during anti-inflammatory therapy. During the development of RA, liver, spleen, and kidney damage of various etiologies can be observed. Serum biochemical analysis showed that the RA model induced obvious fluctuations in hepatic and renal function-related markers, including ALT, AST, CRE, and BUN. After treatment, Leo-micro@MD did not aggravate these biochemical abnormalities. Instead, they showed a clear tendency toward normalization (**Fig. S22**). Organ index analysis further showed no significant differences in the heart, liver, lung, and kidney indexes among the groups, indicating that Leo-micro@MD did not induce detectable organ enlargement or atrophy (**Fig. S23**). Although the spleen index was elevated in the RA model group, consistent with systemic immune activation, both Free Leo and Leo-micro@MD partially reduced this increase. Moreover, H&E staining of the heart, liver, spleen, lung, and kidney revealed no obvious inflammatory infiltration, necrosis, or structural injury after Leo-micro@MD administration (**Fig. S24**). These results indicate that Leo-micro@MD was well tolerated in AIA rats and did not cause apparent systemic toxicity.

## 4 Conclusions

In this work, a long-acting microcomposite depot (Leo-micro@MD) was successfully engineered by encapsulating Leonurine within a buffered PLGA matrix to treat both endometriosis (EMS) and rheumatoid arthritis (RA). Benefiting from internal pH buffering and pore-forming modulation, the system achieved robust drug loading and sustained, minimal initial burst release kinetics extending over 18 days, while pharmacokinetic analysis further supported prolonged systemic Leo exposure *in vivo*. Mechanistically, Leo-micro@MD suppressed cornerstone pro-inflammatory cytokines to branch into distinct therapeutic pathways for both disorders. In EMS, treatment was associated with restoration of the aberrant ERα/PRB balance, enhanced LC3B-related autophagy, and Bax/Bcl-2-associated apoptosis, collectively contributing to ectopic lesion regression. Concurrently, in AIA rats, the platform mitigated synovial inflammation and restricted fibroblast-like synoviocyte invasion, thereby substantially reversing systemic bone erosion and joint damage. Combined with its exceptional biocompatibility and serological safety profiles, this controlled-release strategy provides a powerful, patient-compliant paradigm for the comprehensive management of chronic inflammatory diseases.

## CRediT authorship contribution statement

**Shiyu Meng:** Investigation. **Jingyi Zhong:** Validation, Methodology, Formal analysis. **Yi Xiao:** Validation, Formal analysis. **Xican Zhang:** Methodology, Formal analysis. **Zhuang Tang:** Software, Funding acquisition. **Zhiling Song:** Validation, Methodology. **Xiaoxue Yang:** Methodology. **Yi Zhun Zhu:** Writing – review & editing, Supervision. **Xiaolin Wang:** Writing – review & editing, Supervision, Funding acquisition, Conceptualization. **Hui Guo:** Writing – review & editing, Supervision.

## Ethics declaration

This study was conducted in accordance with the ARRIVE (Animal Research: Reporting of *In Vivo* Experiments) guidelines. This study was approved by the Animal Ethics Committee of Macau University of Science and Technology. (Approval No. MUST-NSFC-20230224001)

## Declaration of competing interest

The authors declare that they have no known competing financial interests or personal relationships that could have appeared to influence the work reported in this paper.

## Data Availability

The data that support the findings of this study are available on request from the corresponding author.
