# Supplementary material for: Leonurine microcomposite depots alleviate endometriosis and rheumatoid arthritis via shared anti-inflammatory cascades
Source: Int J Pharm X. 2026 Oct 17;12:100690. doi: 10.1016/j.ijpx.2026.100690 (PMC13737414; doi:10.1016/j.ijpx.2026.100690)
Supplement: Supplementary file 1 [file mmc1.docx]

Supporting Information for

**Leonurine Microcomposite Depots Alleviate Endometriosis and Rheumatoid Arthritis via Shared Anti-Inflammatory Cascades**

*Shiyu Meng, Jingyi Zhong, Yi Xiao, Xican Zhang, Zhuang Tang, Zhiling Song, Xiaoxue Yang, Yi Zhun Zhu^*^, Xiaolin Wang^*^, Hui Guo^*^*

School of Pharmacy and State Key Laboratory of Mechanism and Quality of Chinese Medicine, Macau University of Science and Technology, Macao 999078, China

*Corresponding author Email:

[yzzhu@must.edu.mo](mailto:yzzhu@must.edu.mo) (Y. Z. Zhu)

[xilwang@must.edu.mo](mailto:xilwang@must.edu.mo) (X. Wang)

[guoh37@mail.sysu.edu.cn](mailto:guoh37@mail.sysu.edu.cn) (H. Guo)

**Supporting Figures**

**Figure S1.** Particle size and zeta potential of Leo-micro. Data were presented as mean ± SD, n = 3.

**Figure S2.** (a) XRD and (b) FT-IR spectra of Leo and Leo-micro.

**Figure S3.** The solubility of Leo-micro in water and PBS (pH 7.4). Data were presented as mean ± SD, n = 3.

**Figure S4.** Chromatographic purity of raw Leo and ball-milled Leo (Leo-micro) determined by HPLC. Data are presented as mean ± SD (n = 3).

**Figure S5.** Preparation of Leo-loaded microspheres by osmotic pressure. Impact of the osmotic pressure on (a) drug loading capability and (b) drug release behavior. Data are presented as mean ± SD, n = 3.

**Figure S6.** Preparation of Leo-loaded microspheres by preparation temperature. Impact of the preparation temperature on (a) drug loading capability and (b) drug release behavior. Data were presented as mean ± SD, n = 3.

**Table S1.** Mathematical modeling of the in vitro release kinetics of Leo-micro@MD

| Model | Equation | R^2^ | AIC |
| --- | --- | --- | --- |
| Zero-order | F = 0.0348t | 0.7869 | −104.12 |
| First-order | F = 1 − exp(-0.0640t) | 0.9888 | −174.84 |
| Higuchi | F = 0.1545t^1/2^ | 0.9652 | −147.58 |
| E | F = 1 − exp[−(t/15.74)^0.905^] | 0.9931 | −184.62 |
| Korsmeyer-Peppas | F = 0.0821t^0.771^ | 0.9812 | — |

**Figure S7.** SEM images of Leo-micro@MD after immersion in PBS at 37°C for 5 weeks. Scale bar = 20 μm.

**Figure S8.** GPC analysis of Leo-micro@MD after immersion in PBS at 37°C for 5 weeks. Data were expressed as mean ± SD, n = 3.

**Figure S9.** Preliminary batch-to-batch reproducibility of the selected Leo-micro@MD formulation. (a) Representative SEM images of three independently prepared batches (Batch 1-3). (b) Loading Efficiency and Encapsulation Efficiency of the three independent batches. 1, 2, 3 represented different batches of samples prepared at different times.Data are presented as mean ± SD. n = 3.

**Figure S10.** Cell viability of (a) primary nESCs and (b) primary eESCs after incubation with different concentrations of Leo-micro@MD. Data were expressed as mean ± SD, n = 3. Statistical significance was calculated by comparing each concentration with the corresponding 0 μM group within the same formulation group. * p < 0.05, **p < 0.01, ***p < 0.001.

**Figure S11.** IL-6 secreted after different treatments. Data were expressed as mean ± SD for n = 3. * p < 0.05, **p < 0.01, ***p < 0.001, ****p < 0.0001.

**Figure S12.** The levels of blood (a) ALT, (b) AST, (c) CRE, and (d) BUN from different groups. Data were presented as mean ± SD for n = 6.

**Figure S13.** Body weight of mice after different treatments over one month. Data were presented as mean ± SD for n = 6.

**Figure S14.** (a) Spleen index and (b) liver index of mice after various treatments. Data were presented as mean ± SD for n = 6.

**Figure S15.** Representative H&E staining images of heart, liver, spleen, lung, and kidney tissue sections from different treatment groups. Scale bar = 100 μm.

**Figure S16.** Cytocompatibility of Leo-micro@MD on MH7A cells after co-incubation for 24 h. Data were expressed as mean ± SD, n = 3.

**Figure S17.** Quantification of IL-10 secreted by RAW 264.7 cells with different treatments. Data were expressed as mean ± SD, n = 3. ** p < 0.01.

**Table S2.** Pharmacokinetic parameters of Leo and Leo-micro@MD in

rats after subcutaneous injection

| Pharmacokinetic parameters | Leo | Leo-micro@MD |
| --- | --- | --- |
| T _max_ (h) | 1.03 ± 0.58 | 1.17 ± 0.41 |
| C _max_ (μg·L⁻¹) | 583.04 ± 150.38 | 508.07 ± 118.21 |
| AUC _0-∞_ (μg·L⁻¹·h) | 1880.30 ± 592.56 | 27812.54 ± 8281.35 |
| T_1/2_ (h） | 1.75 ± 0.45 | 21.66 ± 10.61 |
| MRT _0-∞_ (h) | 2.43 ± 0.41 | 84.37 ± 32.00 |

**Figure S18.** Plasma concentration-time profiles of Leo in rats following subcutaneous administration of Leo and Leo-micro@MD in a period. Data were expressed as mean ± SD, n = 6.

**Figure S19.** Cumulative fraction-input profile following subcutaneous administration of Leo-micro@MD. Numerical deconvolution-derived in vivo input profile of Leo-micro@MD. The plasma concentration–time profile of subcutaneously administered free Leo was used as the immediate-release reference for numerical deconvolution. Fraction input was normalized to the estimated cumulative input at the end of the 8-day.

**Figure 20.** Representative micro-CT images of the ankle joints with different treatments.

**Figure S21.** Histomorphometry parameters were obtained from analyses of the micro-CT data. (a) Total porosity, and (b) SMI: Structure model index. Data are shown as the mean ± SD (n = 6). *p < 0.05, **p < 0.01, ***p < 0.001 and ****p < 0.0001.

**Figure S22.** The level of blood (a) ALT, (b) AST, (c) CRE, and (d) BUN from different groups. Data were presented as mean ± SD for n = 6. *p < 0.05, **p < 0.01, ***p < 0.001 and ****p < 0.0001.

**Figure S23.** (a) Heart index, (b) liver index, (c) spleen index, (d) lung index, and (e) kidney index of mice after various treatments. Data are presented as mean ± SD for n = 6.

**Figure S24.** Representative H&E staining images of heart, liver, spleen, lung, and kidney tissue sections from different treatment groups. Scale bar = 100 μm.
